# Targeting the pentose phosphate pathway mitigates graft-versus-host disease by rewiring alloreactive T cell metabolism

**DOI:** 10.1172/jci.insight.192774

**Published:** 2025-12-08

**Authors:** Saeed Daneshmandi, Eun Ko, Qi Yan, Jee Eun Choi, Prashant K. Singh, Richard M. Higashi, Andrew N. Lane, Teresa W.M. Fan, Jingxin Qiu, Sophia Hani, Keli L. Hippen, Jianmin Wang, Philip L. McCarthy, Bruce R. Blazar, Hemn Mohammadpour

**Affiliations:** 1 Department of Cell Stress Biology, and; 2 Department of Immunology, Roswell Park Comprehensive Cancer Center, Buffalo, New York, USA.; 3 Division of Blood & Marrow Transplant & Cellular Therapy, Department of Pediatrics, University of Minnesota, Minneapolis, Minnesota, USA.; 4 Department of Cancer Genetics & Genomics, Roswell Park Comprehensive Cancer Center, Buffalo, New York, USA.; 5 Department of Toxicology and Cancer Biology, Markey Cancer Center, Center for Environmental and Systems Biochemistry (CESB), Lexington, Kentucky, USA.; 6 Department of Pathology,; 7 Department of Biostatistics & Bioinformatics, and; 8 Department of Medicine, Roswell Park Comprehensive Cancer Center, Buffalo, New York, USA.

**Keywords:** Immunology, Oncology, Stem cell transplantation, T cells

## Abstract

Glycolysis fuels cytotoxic allogeneic T cells in acute graft-versus-host disease (aGvHD), but the downstream role of glucose metabolism in modulating aGvHD remains unclear. Targeting glycolysis or glucose receptors is toxic. Therefore, we explored alternative glucose-dependent pathways, focusing on the pentose phosphate pathway (PPP). Single-cell RNA sequencing revealed PPP upregulation in allogeneic T cells during allogeneic hematopoietic cell transplantation (allo-HCT). We showed that donor T cell deficiency in 6-phosphogluconate dehydrogenase (6PGD), the second rate-limiting enzyme in the PPP, significantly reduced aGvHD severity and mortality in murine models. Functional assays demonstrated that PPP blockade led to proliferation arrest without inducing apoptosis. PPP blockade shifted T cell metabolism away from T cell dependency on glycolysis for rapid T cell proliferation. Pharmacological inhibition of the PPP through 6PGD blockade with 6-aminonicotinamide (6AN) effectively reduced aGvHD severity, like donor 6PGD-deficient T cells in an allogeneic aGvHD model. Similarly, 6AN reduced xenogeneic GvHD lethality. 6PGD inhibition preserved the graft-versus-tumor (GvT) effect, with the generation of a small subset of granzyme B^hi^ effector T cells with potent antitumor activity. These findings highlight the PPP as a key regulator of allogeneic T cell proliferation and differentiation and identify 6PGD as a promising therapeutic target to mitigate aGvHD severity while preserving beneficial GvT effects.

## Abstract 2

After blood stem cell transplants, T cells change metabolism. Blocking a key enzyme, 6PGD, significantly lessens graft-versus-host disease severity without losing the beneficial anti-tumor response.

## Graphical abstract

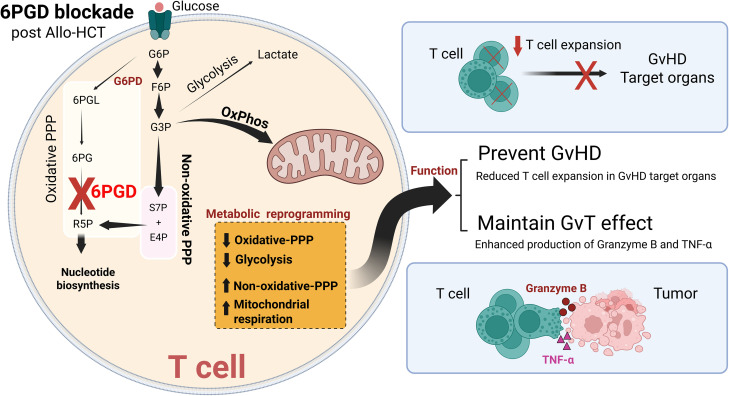

## Introduction

Graft-versus-host disease (GvHD) remains a leading cause of mortality and morbidity following allogeneic hematopoietic cell transplantation (allo-HCT), despite significant advances in therapies aimed at targeting allogeneic T cells (1, 2). Acute GvHD (aGvHD) is predominantly driven by the expansion of helper and cytotoxic T cells, which attack normal host tissues, primarily targeting organs such as the gut, liver, and skin (3, 4). Understanding allogeneic T cell biology in order to develop treatments that can effectively mitigate GvHD severity while preserving graft-versus-tumor (GvT) effects are fundamental for improving allo-HCT outcome.

Allogeneic T cells undergo significant metabolic changes following allo-HCT (5). T cells primarily use glucose as a fuel source, metabolizing it through glycolysis or the pentose phosphate pathway (PPP) (6, 7). Although the role of the T cell glycolysis pathway in aGvHD has been extensively examined (5, 8, 9), little is known about the role of the PPP in allogeneic T cell function.

The PPP, also referred to as the hexose monophosphate shunt, is a critical branch of glycolysis that contributes to nucleotide biosynthesis for energy, and NADPH regeneration and glutathione reduction that buffer oxidants produced during ATP production (10). The PPP pathway consists of a reversible non-oxidative and a largely irreversible oxidative branch (11). The oxidative branch is regulated by enzymes including glucose-6-phosphate dehydrogenase (G6PD), which is allosterically controlled by catalytic products and other metabolites (12). The flux through these branches is dynamically modulated in response to metabolic stress, determining the balance between NADPH and ribose-5-phosphate (R5P) production needed for growth and survival (13). Previous studies have shown that the gene expression of 6-phosphogluconate dehydrogenase (6PGD), the second major rate-limiting enzyme in the PPP, increases in donor T cells in a murine allo-HCT model (5). Our data also reveal that 6PGD is significantly upregulated in allogeneic T cells during the acute phase of GvHD. Because PPP generates nucleotide biosynthesis precursors and can divert glucose metabolism intermediates into the glycolysis pathway, PPP may support GVHD due to the higher need for energy that can be provided by glycolysis (9, 14, 15). In this study, we examined the contribution of the PPP to aGVHD pathobiology, aGvHD severity, efficacy of 6PGD checkpoint pharmacological inhibition, and GvT responses. Our findings offer valuable insights into allogeneic T cell metabolic activity and highlight potential, specific, therapeutic avenues for managing aGvHD while preserving GvT responses.

## Results

### 6PGD gene expression is increased during aGvHD and regulates aGvHD.

**Figure 1:**
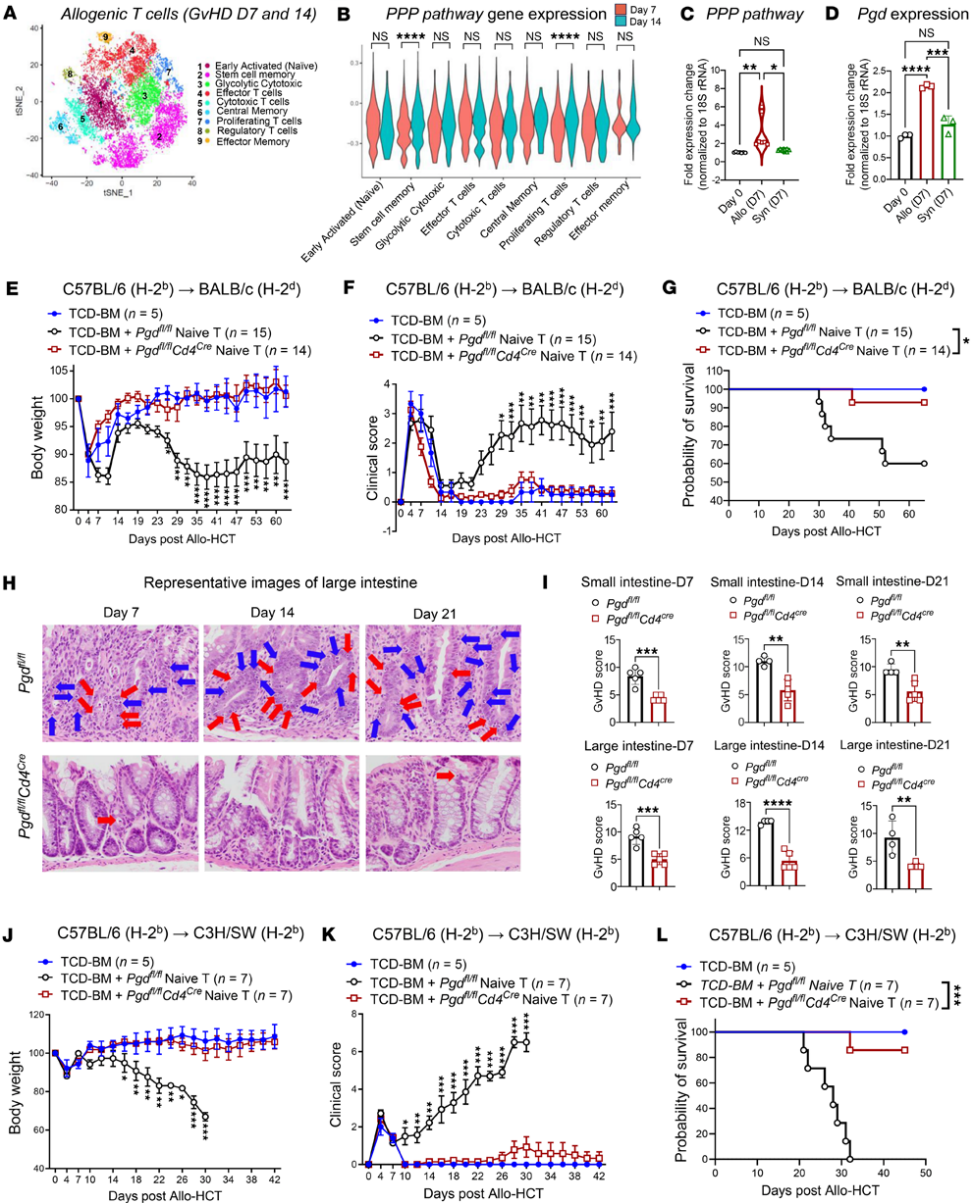
Blockade of oxidative PPP at the 6PGD metabolic checkpoint ameliorates the severity of and the mortality due to aGvHD. (**A** and **B**) BALB/c (H-2^d^) mice were lethally irradiated (8.5 Gy) on day –1 and transplanted with 3.5 × 10^6^ T cell–depleted bone marrow (TCD-BM) cells and 0.7 × 10^6^ purified splenic WT T cells from C57BL/6 (H-2^b^) mice on day 0. Splenic donor T cells (CD45^+^H2K^b+^H2K^d–^TCRβ^+^) were sorted and examined by scRNA-seq (**A**). The expression of oxidative PPP gene signatures was detected within the T cell clusters (**B**). T cells were isolated and pooled for analysis from 5 spleens. (**C** and **D**) Allo-HCT model was generated as in **A** and **B** using 0.2 × 10^6^ purified splenic WT naive T cells. The expression levels of oxidative PPP pathway genes (**C**) and the *Pgd* gene (6PGD) (**D**) were examined in sorted alloreactive T cells (Allo: CD45^+^H2K^b+^H2K^d–^TCRβ^+^) and from BALB/c mice transplanted with syngeneic mice T cells (Syn: CD45^+^H2K^b–^H2K^d+^TCRβ^+^) on day +7. T cells were pooled from 5 spleens (1-way ANOVA). (**E**–**G**) BALB/c (H-2^d^) mice were lethally irradiated (8.5 Gy) on day –1 and transplanted with 3.5 × 10^6^ TCD-BM cells with or without 0.2 × 10^6^ splenic naive T cells from WT (*Pgd^fl/fl^*) or 6PGD-deficient (*Pgd^fl/fl^Cd4^Cre^*) C57BL/6 (H-2^b^) mice on day 0. Body weight loss (**E**) and clinical GvHD scores (**F**) are presented as mean ± SEM. *n* = 5 mice per group (2-way ANOVA). Survival data (**G**) are presented as percentage survival (log-rank Mantel-Cox test). (**H** and **I**) Allo-HCT model was generated as in **E**–**G**. GvHD progression was examined in the GI tract on days +7, +14, and +21. Representative images of H&E staining demonstrate infiltration of intraepithelial lymphocytes (blue arrows) and apoptosis (red arrows) (**H**). Original magnification, ×200. Semiquantitative GvHD scoring of small (top) and large (bottom) intestines are shown. *n* = 5 mice per group (Student’s *t* test). (**J**–**L**) C3H/SW (H-2^b^) mice were lethally irradiated (11.5 Gy) on day –1 and transplanted with 3.5 × 10^6^ TCD-BM cells with or without 0.5 × 10^6^ splenic naive T cells from *Pgd^fl/fl^* or *Pgd^fl/fl^Cd4^Cre^* C57BL/6 (H-2^b^) mice on day 0 as minor-mismatched model. Body weight loss (**J**) and clinical GvHD score (**K**) data are presented as mean ± SEM. Data are representative of 2 independent experiments (2-way ANOVA). Survival data are presented as percentage survival (log-rank Mantel-Cox test) (**L**). Data are shown as mean ± SEM. *P < 0.05; **P < 0.01; ***P < 0.001; ****P < 0.0001.

T cell metabolic reprogramming is entwined with their functional properties (5, 16). Previous studies reported a critical role for PPP after allo-HCT (5). To confirm these findings and to understand T cell metabolic changes after allo-HCT, and how they impact T cell function and allo-HCT outcomes, we employed unbiased single-cell RNA-seq (scRNA-seq) to characterize metabolic changes at days +7 and +14 after allo-HCT. We utilized the fully allogeneic model, C57BL/6→BALB/c, as described above. Donor splenic T cells (CD45^+^H2K^b+^H2K^d–^TCRβ^+^) were sorted on days +7 and +14 for scRNA-seq analysis to identify T cell clusters. Using Seurat analysis, a t-distributed stochastic neighbor embedding (t-SNE) plot revealed 9 T cell clusters (Figure 1A). Proportion analysis showed that naive and stem cell memory T cells were the most abundant, followed by effector/cytotoxic T cells, while central and effector memory T cells represented the smallest fractions (Supplemental Figure 1, A and B; supplemental material available online with this article; https://doi.org/10.1172/jci.insight.192774DS1) (17–22). The pathway enrichment analysis comparing day +14 to day +7 demonstrated upregulation of metabolic pathways, including glucose metabolism and glycolysis, and downregulation of oxidative phosphorylation (OXPHOS) among top 10 pathway changes as aGvHD progresses (Supplemental Figure 1C). Comparative analysis indicated elevated PPP gene signatures in stem cell memory T cells and proliferating clusters on day +14 when compared with day +7 (Figure 1B). An analysis of oxidative PPP during aGvHD initiation using *G6pdx*, *G6pd2*, *Pgd*, and *Pgls* gene sets revealed increased expression of oxidative PPP genes on day +7 when compared with day 0 for allogeneic, but not syngeneic, T cells (Figure 1C), particularly *Pgd* (which encodes 6PGD). This was confirmed by real-time PCR (Figure 1D). Western blot analysis further validated the enhanced 6PGD expression in alloreactive T cells (Supplemental Figure 2, A and B).

To explore the role of PPP, specifically 6PGD as a metabolic checkpoint regulating T cell function during aGvHD, we developed *Cd4^Cre^* × *Pgd^fl/fl^* mice, as described in the Methods. In these mice, 6PGD is inactivated in both CD4^+^ and CD8^+^ T cells since Cre is expressed at the CD4^+^CD8^+^ thymic development stage (15, 23).

We sought to determine the intrinsic impact of 6PGD deficiency on the resting splenic T cell phenotypes. Compared with WT controls, 6PGD-KO mice exhibited a lower frequency of CD4^+^ and CD8^+^ T cell subsets (Supplemental Figure 3A). Differentiation marker analysis revealed an increased frequency of effector T cells (CD62L^–^CD44^+^) among CD4^+^ (Supplemental Figure 3B) and CD8^+^ (Supplemental Figure 3C) populations. Resting-state 6PGD-KO T cells demonstrated a heightened expression of inflammatory cytokines, including IFN-γ (Supplemental Figure 3, D and E), TNF-α (Supplemental Figure 3, F and G), and GM-CSF (Supplemental Figure 3, H and I) when compared with WT T cells.

We therefore utilized purified naive T cells as donor T cells for the allo-HCT experiments to test the role of the PPP at the 6PGD checkpoint on aGvHD severity and mortality. We flow sorted naive T cells and found comparable CD4^+^ and CD8^+^ populations between the *Pgd^fl/fl^Cd4^Cre^* and *Pgd^fl/fl^* T cells with high purity (Supplemental Figure 4). To evaluate T cell 6PGD deficiency during aGvHD, WT BALB/c mice were irradiated (8.5 Gy with ^137^Cs) and transplanted i.v. with 3.5 × 10^6^ total cells of T cell–depleted bone marrow (TCD-BM) from C57BL/6 mice with or without 0.2 × 10^6^ naive T cells isolated from *Pgd^fl/fl^* or *Pgd^fl/fl^Cd4^Cre^* C56BL/6 mice. Compared with recipients of WT T cells, transplanted recipients of 6PGD-KO donor T cells showed reduced aGvHD severity, evidenced by decreased body weight loss (Figure 1E), lower clinical GvHD scores (Figure 1F), and improved survival (Figure 1G). As expected, recipients of TCD-BM alone did not develop aGvHD (Figure 1, E–G).

Histopathological examination of recipient target organs (small and large intestines) confirmed the clinical and survival data. Using a semiquantitative scoring system (24, 25), evaluator-blinded analysis of H&E-stained sections on days +7, +14, and +21 demonstrated lower aGvHD scores in recipients of *Pgd^fl/fl^Cd4^Cre^* T cells when compared with WT T cells, with reduced intraepithelial lymphocyte infiltration (blue arrows) and apoptosis (red arrows; nuclear and cytoplasm condensation) (Figure 1H). GI histopathology at lower and higher magnification on day +21 and liver histopathology on day +7 and day +14 are shown in Supplemental Figure 5. Semiquantitative scoring confirmed these observations (Figure 1I). Additionally, our previous reports showed the key role of 6PGD on regulation of endogenous (autologous) CD8^+^ T cell function (15). To examine the role of the PPP in CD8^+^ T cell–induced aGvHD, WT BALB/c mice were irradiated (8.5 Gy with ^137^Cs) and transplanted i.v. with 3.5 × 10^6^ TCD-BM cells (from C57BL/6 mice) with or without 1 × 10^6^ naive CD8^+^ T cells isolated from *Pgd^fl/fl^* or *Pgd^fl/fl^Cd4^Cre^* C56BL/6 mice. The results indicate that, like total T cells, 6PGD-KO donor CD8^+^ T cells showed reduced aGvHD severity, evidenced by decreased body weight loss (Supplemental Figure 6A), lower clinical GvHD scores (Supplemental Figure 6B), and improved survival (Supplemental Figure 6C).

To confirm the effects of 6PGD blockade in donor T cells in an MHC-matched, minor antigen–mismatched model, irradiated C3H/SW recipients were given 0.5 × 10^6^ splenic naive T cells from either *Pgd^fl/fl^Cd4^Cre^* or *Pgd^fl/fl^* C57BL/6 donors, along with 3.5 × 10^6^ TCD-BM cells (from C57BL/6 mice). C3H/SW recipients of 6PGD-KO T cells when compared with WT T cells displayed reduced body weight loss (Figure 1J), lower clinical GvHD scores (Figure 1K), and improved survival (Figure 1L) compared with controls.

### Blockade of the 6PGD metabolic checkpoint prevents early expansion of allogeneic T cells while preserving a subset with an effector phenotype.

**Figure 2:**
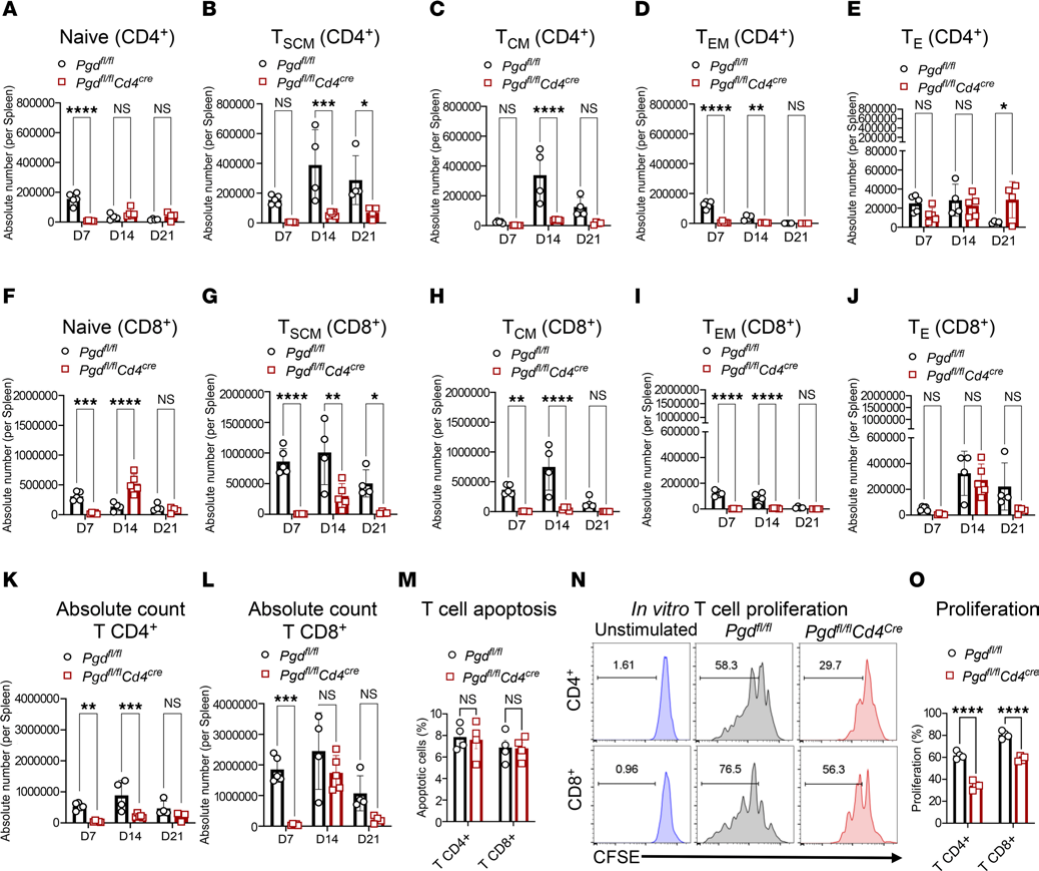
Blockade of the 6PGD metabolic checkpoint prevents alloreactive T cell expansion while maintaining a portion of T cells with effector phenotype. (**A**–**J**) BALB/c (H-2^d^) mice were lethally irradiated (8.5 Gy) on day –1 and transplanted with 3.5 × 10^6^ TCD-BM cells with or without 0.2 × 10^6^ splenic naive T cells from *Pgd^fl/fl^* or *Pgd^fl/fl^Cd4^Cre^* C57BL/6 (H-2^b^) mice on day 0. The frequency of CD4^+^ T cells subsets as naive (**A**), stem cell memory (**B**), central memory (**C**), effector memory (**D**), and effector (**E**) was detected per spleen of the mice. The frequency of CD8^+^ T cells subsets as naive (**F**), stem cell memory (**G**), central memory (**H**), effector memory (**I**), and effector (**J**) was determined per spleen of the mice. *n* = 4–5 mice per group. Two-way ANOVA was used to analyze statistical significance among groups. The absolute number of CD4^+^ (**K**) and CD8^+^ (**L**) T cells was calculated per spleen on days +7, +14, and +21. *n* = 4–5 mice per group (2-way ANOVA). (**M**) T cells were isolated from *Pgd^fl/fl^* or *Pgd^fl/fl^Cd4^Cre^* spleens and activated in vitro with plate-bound anti-CD3 plus anti-CD28 mAbs. Apoptosis was determined by Annexin V staining. *n* = 4 data points per group. Data representative of 2 independent experiments (Student’s *t* test). (**N** and **O**) T cells were isolated from *Pgd^fl/fl^* or *Pgd^fl/fl^Cd4^Cre^* spleens, labeled with CFSE, and activated in vitro with plate-bound anti-CD3 plus anti-CD28 mAbs. The proliferation of CD4^+^ (top) and CD8^+^ (bottom) T cells was examined after 72 hours. Unstimulated T cells served as negative controls. *n* = 3 data points per group. Data representative of 3 independent experiments (Student’s *t* test) and are shown as mean ± SEM. *P < 0.05; **P < 0.01; ***P < 0.001; ****P < 0.0001.

We sought to understand possible mechanisms as to how 6PGD deficiency in donor naive T cells significantly reduces the severity and mortality of aGvHD. Using the MHC-mismatched (fully allogeneic) aGvHD model (C57BL/6 to BALB/c), we evaluated the impact of 6PGD deficiency on T cell subset dynamics, proliferation, and effector function. Flow cytometric analysis of splenic and hepatic donor T cells in recipients on days +7, +14, and +21 after allo-HCT revealed reduction in the frequencies of various CD4^+^ and CD8^+^ T cell subsets, including naive (Figure 2, A and F), stem cell memory (Figure 2, B and G), central memory (Figure 2, C and H), and effector memory (Figure 2, D and I). However, the frequency of effector CD4^+^ T cells increased, while no significant changes were observed in the effector CD8^+^ T cell population (Figure 2, E and J). The absolute CD4^+^ and CD8^+^ T cell numbers were significantly lower in 6PGD-deficient groups (Figure 2, K and L). Similar trends were observed when evaluating T cell subtype frequencies as percentages of total T cells within individual groups (Supplemental Figure 7). T cell subsets were identified according to the established markers in previous publications (26–29).

**Figure 3:**
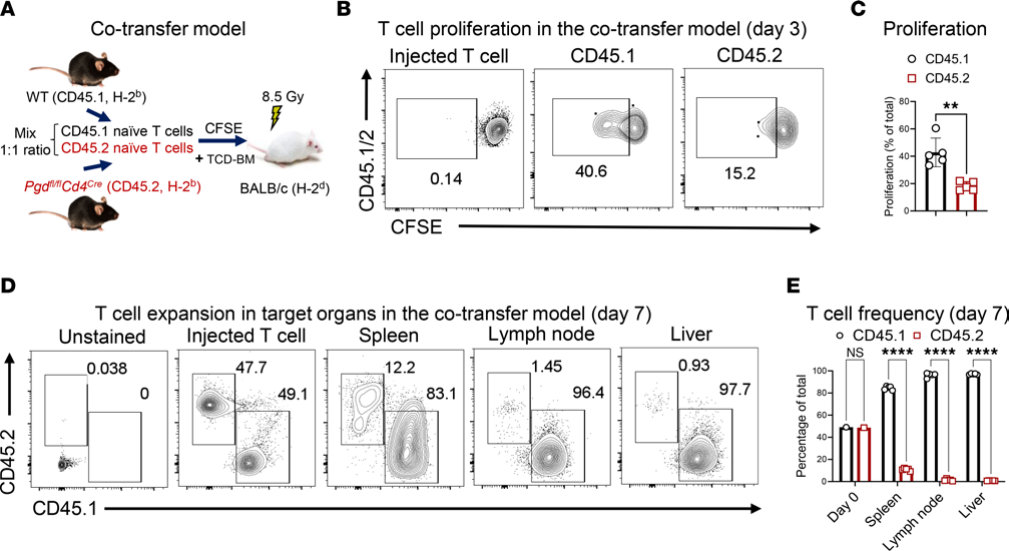
6PGD blockade prevents early expansion of alloreactive T cells in aGvHD models. (**A**–**C**) Naive T cells were isolated from CD45.1^+^ WT and CD45.2^+^ *Pgd^fl/fl^Cd4^Cre^* mice, mixed 1:1, and labeled with CFSE. TCD-BM cells (3.5 × 10^6^) and 0.4 × 10^6^ mixed CSFE-labeled T cells infused into lethally irradiated (8.5 Gy) BALB/c mice (**A**). Proliferation was determined on day +3 by CFSE dilution in splenic CD45.1^+^ and CD45.2^+^ T cells (**B** and **C**). *n* = 4–5 mice per group. Data represent one independent experiment (Student’s *t* test). (**D** and **E**) Isolated naive T cells from CD45.1^+^ WT and CD45.2^+^ *Pgd^fl/fl^Cd4^Cre^* mice were mixed 1:1. TCD-BM cells (3.5 × 10^6^) and 0.4 × 10^6^ mixed T cells were infused into lethally irradiated (8.5 Gy) BALB/c mice. The frequencies of CD45.1^+^ and CD45.2^+^ T cells in spleen, mesenteric lymph node, and liver were examined on day +7 after transplantation (**D**). The comparative frequencies of CD45.1^+^ and CD45.2^+^ T cells are given as the percentage of total T cells (**E**). Day 0 is the time of cell injection. *n* = 4–5 mice per group. Data represent 1 experiment (Student’s *t* test). Data are shown as mean ± SEM. **P < 0.01; ****P < 0.0001.

We sought to determine whether the reduced T cell numbers were due to increased apoptosis or impaired proliferation. T cells were isolated from resting *Pgd^fl/fl^* and *Pgd^fl/fl^Cd4^Cre^* C57BL/6 mouse spleens and stimulated in vitro with anti-CD3 and anti-CD28 mAbs for 72 hours. Comparable viability in both groups was determined by Annexin V staining (Figure 2M). However, 6PGD-deficient T cells exhibited significantly lower proliferation (Figure 2, N and O). C57BL/6 donor T cell proliferation was assessed in vivo using a CFSE dilution assay with a 1:1 mixture of CD45.1^+^ WT and CD45.2^+^ *Pgd^fl/fl^Cd4^Cre^* naive T cells (0.2 × 10^6^ total cells), transferred into lethally irradiated BALB/c mice. On day +3, CFSE dilution analysis confirmed that 6PGD-KO (CD45.2^+^) T cells proliferated significantly less compared with WT T cells (Figure 3, A–C). Evaluation of donor T cell expansion in spleen, mesenteric lymph nodes, and liver on day +7 showed significantly lower frequencies of CD45.2^+^ 6PGD-deficient T cells compared with CD45.1^+^ WT T cells (Figure 3, D and E). Time of cell injection is referred to as day 0 in these experiments.

### Pharmacological blockade of 6PGD with 6AN reduces GvHD severity in murine aGvHD and xenogeneic aGvHD models.

**Figure 4:**
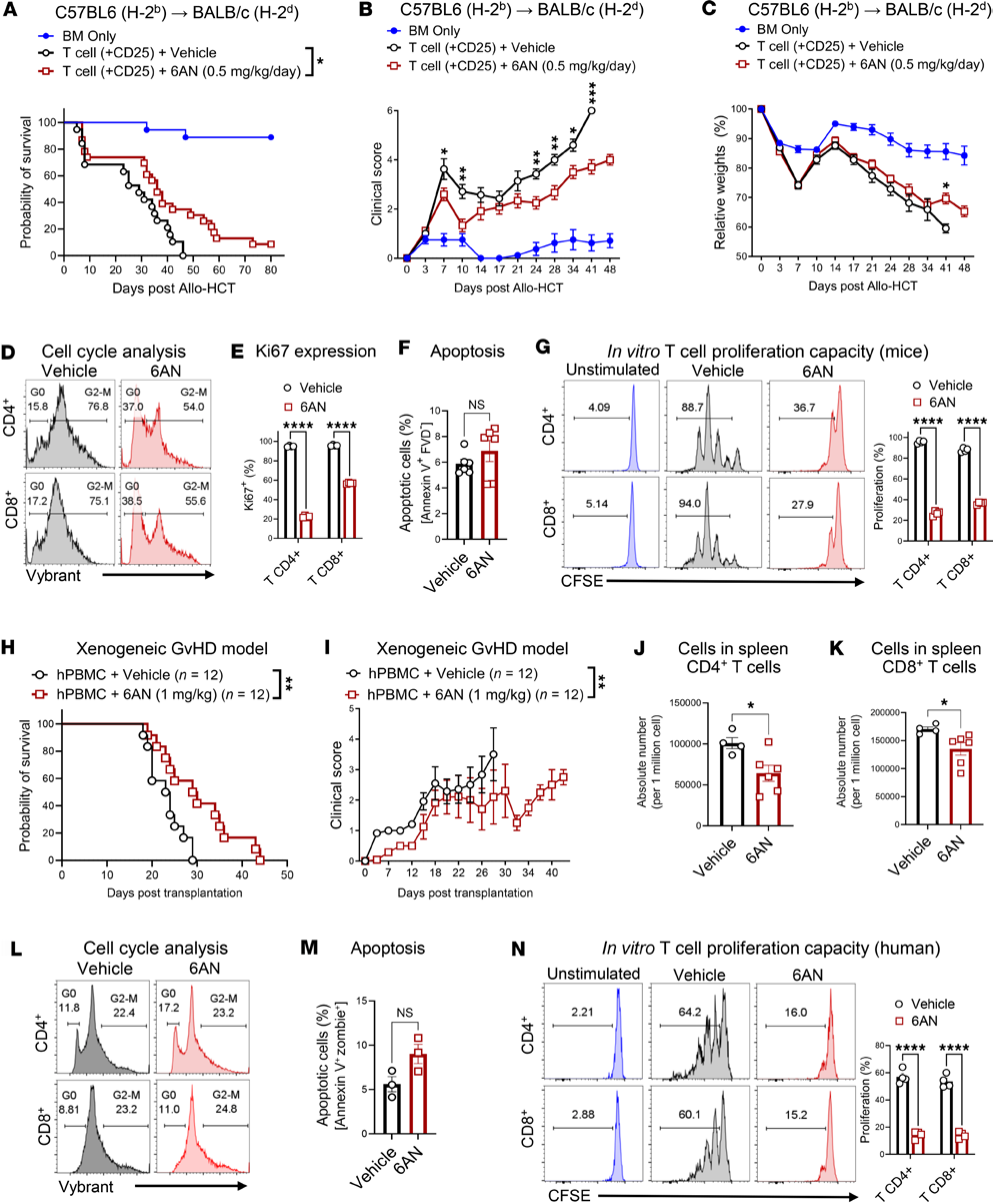
6-Aminonicotinamide (6AN), a small-molecule inhibitor of 6PGD, reduces GvHD severity in a fully allogeneic murine model and a xenogeneic GvHD model. (**A**–**C**) BALB/c (H-2^d^) mice were lethally irradiated (6 Gy) on day –1 and transplanted i.v. with 1 × 10^7^ BM cells with or without 1 × 10^6^ splenic T cells from WT C57BL/6 (H-2^b^) mice on day 0. Mice were injected i.p. with 0.5 mg/kg 6AN or vehicle (1% DMSO) daily. Mouse survival (**A**), GvHD clinical score (**B**), and body weight loss (**C**) are reported. *n* = 18–20 mice per group combined from 3 independent repeats (2-way ANOVA). (**D**) Splenic and lymph node T cells from WT C57BL/6 mice were activated in vitro with plate-bound anti-CD3 and anti-CD28 (10 μg/mL each) plus rmIL-2 (100 ng/mL) for 72 hours and examined by Vybrant DyeCycle (catalog V35003, Thermo Fisher Scientific) for cell cycle determination. (**E** and **F**) T cells were treated as in **D** and examined for Ki67 proliferation marker (**E**) and apoptosis rate (**F**). *n* = 3–6 data points per group. Data representative of 3 independent repeats (Student’s *t* test). (**G**) T cells were treated as in **D** after CFSE labeling and proliferation of CD4^+^ (top) and CD8^+^ (bottom) T cells was examined after 72 hours. *n* = 3 data points per group. Data representative of 3 independent repeats (Student’s *t* test). (**H** and **I**) NSG mice were sublethally irradiated (2.5 Gy) on day –1 and transplanted with 2 × 10^6^ human PBMCs on day 0. Mice were injected i.p. with 1 mg/kg 6AN or vehicle every 2 days. Mouse survival (**H**) and GvHD clinical scores (**I**) are reported. *n* = 12 mice per group from 2 independent repeats (2-way ANOVA). (**J** and **K**) Mice were transplanted as in **H** and **I** and numbers of splenic CD4^+^ (**J**) and CD8^+^ (**K**) T cells were examined on day +14 after allo-HCT (Student’s *t* test). (**L** and **M**) Human T cells from healthy donors were activated with anti-CD3 and anti-CD28 (10 μg/mL each) plus rhIL-2 (100 ng/mL) and treated with 6AN or vehicle for 72 hours. Cell cycle status was determined by Vybrant DyeCycle (**L**) and apoptosis rate by Annexin V staining (**M**). *n* = 3 data points per group. Data representative of 2 independent repeats (Student’s *t* test). (**N**) Human T cells were isolated and treated as in **L** after CFSE labeling. The proliferation of CD4^+^ (top) and CD8^+^ (bottom) T cells was examined after 72 hours. *n* = 3 data points per group. Data representative of 2 independent repeats (Student’s *t* test). Data are shown as mean ± SEM. *P < 0.05; **P < 0.01; ***P < 0.001; ****P < 0.0001.

To assess whether pharmacological inhibition of 6PGD could mitigate aGvHD lethality, we tested 6-aminonicotinamide (6AN), a small-molecule inhibitor of 6PGD, in both murine and xenogeneic GvHD models. In the C57BL/6→BALB/c aGvHD model, 6AN treatment (0.5 mg/kg, administered daily via i.p. injection starting on day 0 of allo-HCT for the experiment duration) significantly improved long-term survival, with 35% survival of the treated group on day +46 after allo-HCT compared with 0% survival in controls, with reduced clinical GvHD scores and improved weight loss after day +41 (Figure 4, A–C). In vitro studies indicated that 6AN impairs T cell proliferation, which is associated with disrupted cell-cycle progression. 6AN-treated T cells showed a 12%–18% reduction in the proportion of cells in the G_2_/M phase (Figure 4D) and a corresponding lower fraction of proliferating Ki67^+^ cells (Figure 4E). Importantly, 6AN treatment did not increase the preapoptotic Annexin V^+^ fixable viability dye–negative (FVD^–^) population (Figure 4F), indicating that the reduction in T cell proliferation is independent of increased cell death during the 72 hours of incubation. Instead, 6AN specifically inhibited proliferation (Figure 4G). These findings favor the hypothesis that 6PGD deficiency mitigates T cell proliferation, protecting against severe aGvHD.

To evaluate the translational potential of 6AN, we employed a xenogeneic GvHD model using NSG mice. Recipients were sublethally irradiated (2.5 Gy, ^137^Cs source, day –1) and transplanted i.v. with 2 × 10^6^ human PBMCs on day 0. Mice were treated 6AN or vehicle (1% DMSO) every 2 days (1 mg/kg), given every 2 days via i.p. injection beginning on day 0 of allo-HCT for the experiment duration. 6AN-treated recipients had significantly improved survival (Figure 4H; median survival of 23.5 days in the control group vs. 29.5 days in treated group, *P* = 0.008) and reduced clinical GvHD scores (Figure 4I). Analysis of donor human CD4^+^ and CD8^+^ T cells in treated mice revealed a significant reduction in cell numbers compared with vehicle-treated controls (Figure 4, J and K). Most T cell subsets were significantly reduced except for CD8^+^ effector memory T and CD4^+^ and CD8^+^ effector T cells (Supplemental Figure 8).

In vitro treatment of human T cells with 6AN led to a reduction in cell cycle progression (Figure 4L), without affecting cell viability (Figure 4M). 6AN-treated human T cells had a significant impairment in proliferation (Figure 4N), consistent with the murine in vitro results shown in Figure 4, D–F. The reduced capacity for donor T cell proliferation correlated with decreased alloreactivity and lower GvHD severity. In aggregate, the 6AN results highlight the potential of 6PGD inhibition as a therapeutic strategy for managing GvHD.

### 6PGD deficiency in T cells induces metabolic reprogramming toward reduced glycolysis and enhanced mitochondrial respiration.

**Figure 5:**
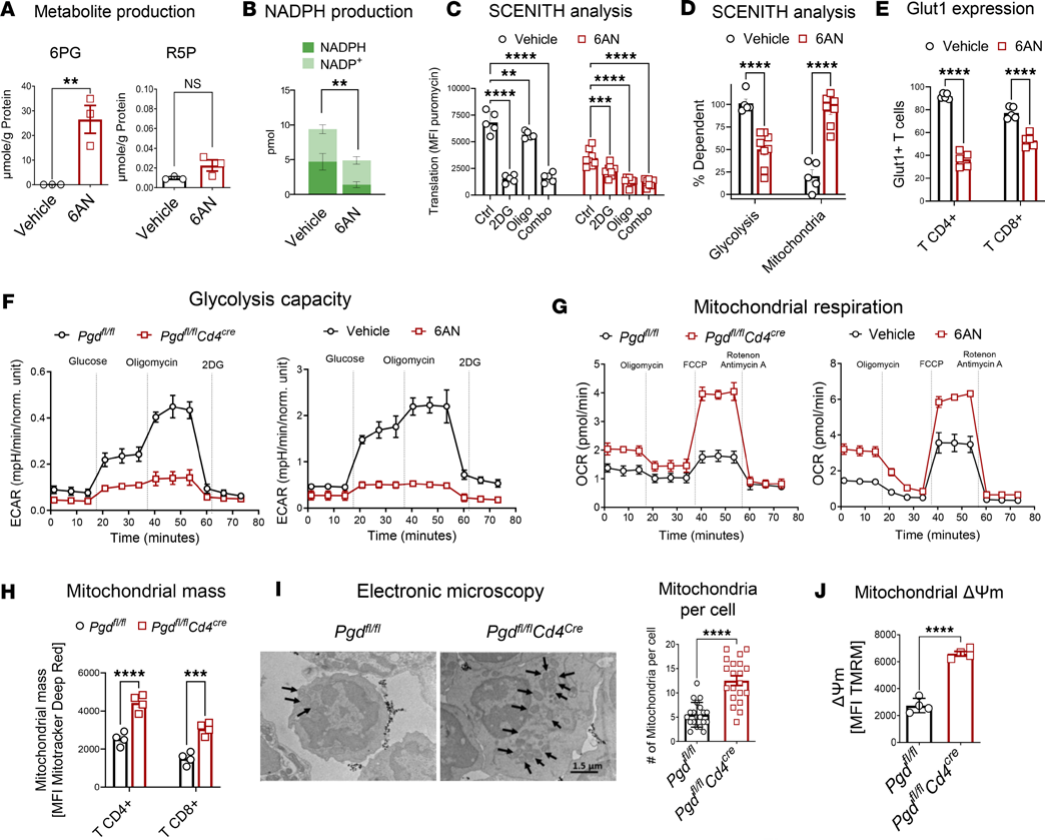
6PGD blockade in T cells induces a metabolic reprogramming evident by reduced glycolysis and higher mitochondrial respiration. (**A**) Splenic naive T cells from WT C57BL/6 mice were activated in vitro with plate-bound anti-CD3 and anti-CD28 mabs (10 μg/mL each) plus rmIL-2 (100 ng/mL) for 4 days with 6AN (10 μM) or vehicle (DMSO) control, and then exposed to 10 mM D_7_-D-glucose. The 6-phosphogluconate (6PG) and ribose-5-phosphate (R5P) quantities generated from glucose were determined at 8 hours. *n* = 3 data points per group (Student’s *t* test). (**B**) Levels of NADPH and NADP^+^ in in vitro–activated T cells after 6AN or vehicle treatment for 72 hours are shown. *n* = 3 data points per group (Student’s *t* test). (**C** and **D**) The metabolic profiles of 6AN- and vehicle-treated T cells were analyzed by SCENITH single-cell translation profiling (**C**). 6AN-treated T cells showed lower glycolysis and higher mitochondrial function when compared with vehicle (**D**). *n* = 4–5 data points per group. Data representative of 2 independent repeats (2-way ANOVA). (**E**) In vitro–activated T cells in presence of 6AN or vehicle were examined for expression of the plasma membrane glucose transporter Glut1. *n* = 4–5 data points per group. Data representative of 2 independent repeats (Student’s *t* test). (**F** and **G**) Splenic naive T cells from *Pgd^fl/fl^* or *Pgd^fl/fl^Cd4^Cre^* mice were activated with plate-bound anti-CD3 and anti-CD28 (10 μg/mL each) plus rmIL-2 (100 ng/mL) for 4 days. For the pharmacologic experiment, naive T cells were isolated from WT mice and stimulated as above in the presence of 6AN (10 μM) or vehicle control. The harvested cells were examined for extracellular acidification rate (ECAR) (**F**) or oxygen consumption rate (OCR) (**G**) using the Seahorse assay. *n* = 4 data points per group. Data representative of 2 independent repeats. (**H**) T cells were prepared and activated as in **F** and **G**. The mitochondrial mass was determined by MitoTracker Deep Red staining. *n* = 4 data points per group. Data representative of 3 independent repeats (Student’s *t* test). (**I** and **J**) T cells were prepared as in **F** and **G** and examined by transmission electron microscopy. The mitochondria in the cells are highlighted by black arrows and mitochondrial number was counted in 20 random fields for each group (**I**). Scale bar: 1.5 μm. Mitochondrial activity was examined by evaluation of mitochondrial potential (ΔΨm) by tetramethylrhodamine ester (TMRE) (**J**). *n* = 4 data points per group. Data representative of 3 independent repeats (Student’s *t* test) and are shown as mean ± SEM. *P < 0.05; **P < 0.01; ***P < 0.001; ****P < 0.0001.

Isotope tracing precisely tracks cellular metabolic cascades (30). To determine the specificity of 6PGD blockade and understand the fate of glucose during 6PGD inhibition, we employed stable isotope–resolved metabolomics (SIRM) using D_7_-D-glucose labeling. Splenic naive T cells were activated with anti-CD3/anti-CD28 monoclonal antibodies and recombinant mouse IL-2 (rmIL-2), with or without 6AN treatment (Figure 5A). In 6AN-treated T cells, there was accumulation of 6-phosphogluconate (6PG), the substrate of 6PGD, confirming enzymatic blockade (Figure 5A). However, levels of R5P, a critical PPP product required for nucleotide biosynthesis, were not affected when compared to vehicle (DMSO) (Figure 5A). Instead, the non-oxidative arm of the PPP was upregulated, leading to increased intermediate levels of erythrose-4-phosphate (E4P), fructose-6-phosphate (F6P), and glyceraldehyde-3-phosphate (G3P) (Supplemental Figure 9). These results confirm that 6PGD blockade occurs following 6AN treatment and that the non-oxidative PPP is upregulated as a compensatory mechanism, providing R5P production for nucleotide biosynthesis.

One of the effects of the PPP is NADPH production (12). To further confirm the impact of 6PGD blockade, we measured NADPH levels in T cells. As expected, NADPH production was significantly reduced in 6AN-treated cells, the functional consequence of 6PGD inhibition (Figure 5B).

To explore how 6PGD blockade alters the metabolic capacity of T cells, we performed SCENITH single-cell translation profiling (31), a flow cytometry–based method to assess energy metabolism. This revealed reduced overall metabolic capacity in 6AN-treated T cells, consistent with shifting to a resting phenotype (Figure 5C). SCENITH analysis showed that the glucose analog 2-deoxy-D-glucose (2DG) significantly decreased glycolysis, while oligomycin, which blocks OXPHOS by inhibiting membrane-bound mitochondrial ATP synthetase, had a significant but modest impact on WT T cells, consistent with a strong reliance of WT T cells on glycolysis. Conversely, in 6AN-treated T cells, the inhibition of OXPHOS using oligomycin had the most significant impact on T cell metabolism, indicating that these T cells rely on OXPHOS for their energy metabolism (Figure 5C). Metabolic profiling further demonstrated a shift from glycolysis to mitochondrial respiration, with decreased glucose dependency and enhanced mitochondrial activity (Figure 5D). This metabolic reprogramming was accompanied by a reduced surface expression of the glucose transporter Glut1, which facilitates glucose transport across the plasma membrane (Figure 5E). Seahorse flux analysis confirmed these findings, showing lower glycolytic activity and increased mitochondrial respiration in T cells without 6PGD in *Pgd^fl/fl^Cd4^Cre^* mice or WT mice given 6AN for 6PGD pharmacologic blockade (Figure 5, F and G).

As noted above, mitochondrial function is enhanced in 6PGD-KO (*Pgd^fl/fl^Cd4^Cre^*) T cells. These cells exhibited increased mitochondrial mass and mitochondrial numbers per cell, with elevated mitochondrial membrane potential (Figure 5, H–J). 6PGD blockade reprograms T cell metabolism to prioritize the non-oxidative PPP for nucleotide precursor production (Supplemental Figure 9), while shifting energy generation from glycolysis to mitochondrial respiration (Figure 5).

Previous reports showed the importance of glycolysis in donor T cell survival (32). Our studies confirm that 6PGD blockade does not induce T cell death in the allo-HCT model in vivo (Supplemental Figure 10, A and B) or in T cells activated in vitro as early as 24 hours after stimulation (Supplemental Figure 10, C–E). These results suggest that 6PGD blockade reprograms T cell metabolism to switch from glycolysis to mitochondrial respiration, providing energy for T cell survival and function. The metabolic shift decreases T cell proliferation through diminished glycolysis and reprograms T cells toward a resting state, contributing to the reduced severity and mortality of aGvHD. Thus, 6PGD is central to the integration of metabolic pathways to regulate T cell function and may serve as a therapeutic target.

### 6PGD blockade preserves GvT responses while ameliorating aGvHD.

**Figure 6:**
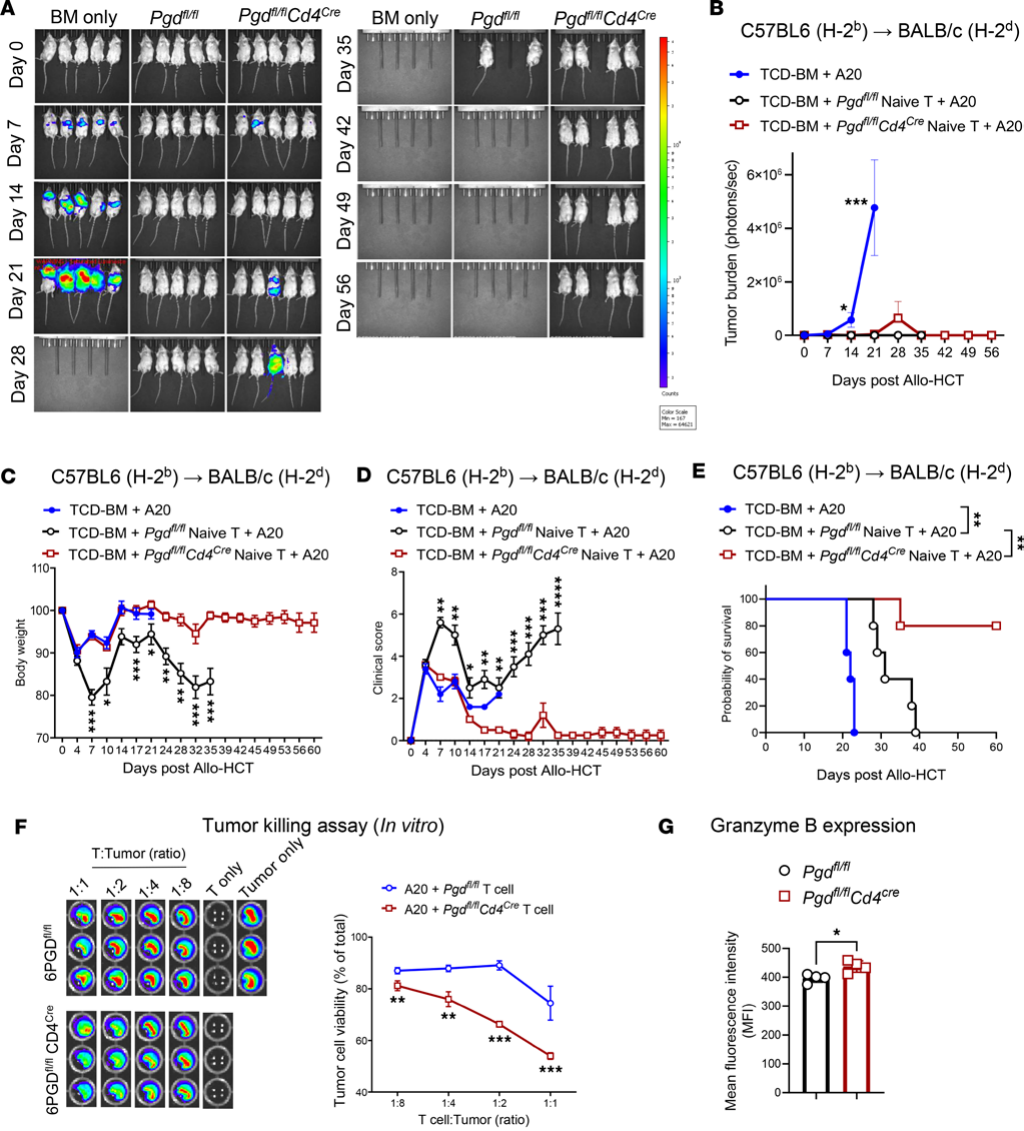
6PGD blockade preserved GvT responses while ameliorating aGvHD. (**A** and **B**) BALB/c (H-2^d^) mice were lethally irradiated (8.5 Gy) on day –1 and transplanted i.v. with 3.5 × 10^6^ TCD-BM cells with or without 0.2 × 10^6^ splenic naive T cells from *Pgd^fl/fl^* or *Pgd^fl/fl^Cd4^Cre^* C57BL/6 (H-2^b^) mice plus 0.1 × 10^6^ A20-Luc^+^ tumor cells on day 0. Tumor growth was monitored by bioluminescence imaging (BLI) (**A**). Tumor growth was quantified by detection of bioluminescent signals (**B**). *n* = 5 mice per group. Data representative of 2 independent repeats (2-way ANOVA). (**C**–**E**) In the mice treated as in **A** and **B**, body weight loss (**C**) and clinical GvHD score (**D**) were measured and are presented as mean ± SEM. Two-way ANOVA was used to analyze statistical significance among groups. (**E**) Survival of mice was monitored and is presented as percentage survival. *n* = 5 mice per group. Data representative of 2 independent repeats (log-rank Mantel-Cox test). (**F**) For tumor killing by activated T cells, splenic naive T cells were isolated from *Pgd^fl/fl^* or *Pgd^fl/fl^Cd4^Cre^* mice and activated with plate-bound anti-CD3 and anti-CD28 (10 μg/mL each) plus rmIL-2 (100 ng/mL) for 4 days. Harvested T cells were cocultured with A20-Luc^+^ tumor cells and tumor killing was measured after 18 hours using BLI. Tumor cell survival was quantified by detection of bioluminescent signals. *n* = 3 time points per group. Data representative of 3 independent repeats (2-way ANOVA). (**G**) T cells were activated as in **F**. T cell granzyme B expression was detected by flow cytometry. *n* = 3 time points per group. Data representative of 3 independent repeats (Student’s *t* test) and are shown as mean ± SEM. *P < 0.05; **P < 0.01; ***P < 0.001; ****P < 0.0001.

The reduction in T cell proliferation in 6PGD-KO T cells led us to investigate their capacity to sustain the GvT effect to control malignancy. To evaluate the effect of 6PGD blockade, BALB/c recipients underwent lethal irradiation (8.5 Gy, ^137^Cs, day –1). On day 0, lethally irradiated mice were injected with 0.1 × 10^6^ luciferase-expressing A20 (A20-Luc^+^) BALB/c tumor cells 4 hours before transplantation with 3.5 × 10^6^ TCD-BM cells from C57BL/6 WT mice and 0.2 × 10^6^ splenic naive T cells from *Pgd^fl/fl^* or *Pgd^fl/fl^Cd4^Cre^* donors. Controls receiving only tumor cells and TCD-BM exhibited significant tumor growth by bioluminescence imaging (BLI) (Figure 6, A and B). Tumor growth was controlled in recipients of T cells from both *Pgd^fl/fl^* and *Pgd^fl/fl^Cd4^Cre^* donors, indicating that 6PGD-KO T cells retained GvT activity (Figure 6, A and B). *Pgd^fl/fl^Cd4^Cre^* T cell recipients also exhibited reduced aGvHD severity compared with those receiving *Pgd^fl/fl^* T cells, as demonstrated by improved body weight (Figure 6C), lower clinical GvHD scores (Figure 6D), and enhanced survival (Figure 6E). These results indicated that 6PGD blockade in donor T cells achieves the dual benefit of mitigating aGvHD by inhibiting the T cells, which causes GvHD while preserving the GvT efficacy even with a reduction in cytotoxic T cells.

To confirm the antitumor capacity of 6PGD-deficient T cells, splenic naive T cells were isolated from *Pgd^fl/fl^* or *Pgd^fl/fl^Cd4^Cre^* mice, activated in vitro with anti-CD3/anti-CD28 mAbs, and cocultured with A20-Luc^+^ tumor cells. Tumor killing was assessed at 18 hours using BLI based on our previous publication (24, 33). *Pgd^fl/fl^Cd4^Cre^* T cells exhibited enhanced tumor-killing capacity compared with *Pgd^fl/fl^* T cells (Figures 6F). Increased cytotoxicity of 6PGD-deficient T cells against A20-Luc+ tumor cell was associated with higher granzyme B expression (Figure 6G), a known factor for T cell-mediate A20-Luc+ tumor cell lysis (34). Previous studies reported that generation of lactic acid by tumor cells disrupts the metabolic fitness of T cells, including reduced glycolysis, resulting in defective T cell antitumor responses (32). Our results suggest that 6PGD blockade reprograms T cell metabolism to switch from glycolysis to mitochondrial respiration. This metabolic switch prevents adverse effects (toxicity) while inducing a tumor-killing phenotype in T cells.

**Figure 7:**
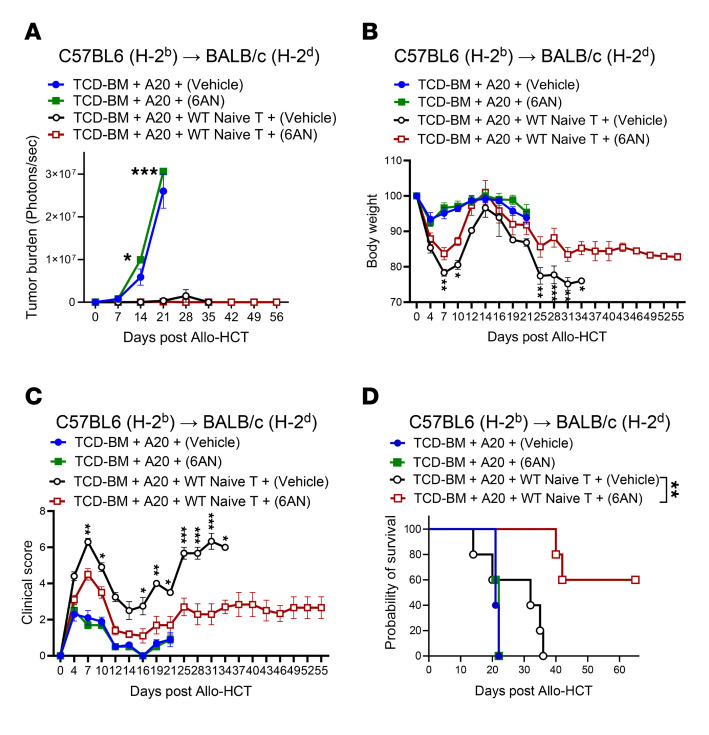
Pharmacological inhibition of 6PGD suppresses aGvHD while maintaining GvT responses. (**A**–**D**) BALB/c (H-2^d^) mice were lethally irradiated (8.5 Gy) on day –1 and transplanted i.v. with 3.5 × 10^6^ TCD-BM cells with or without 0.2 × 10^6^ splenic naive T cells from WT C57BL/6 (H-2^b^) mice plus 0.1 × 10^6^ A20-Luc^+^ tumor cells on day 0. Mice were injected i.p. with 0.5 mg/kg 6-aminonicotinamide (6AN) or vehicle (1% DMSO) daily. Tumor growth was quantified by detection of bioluminescent signals (**A**). Treated mice body weight loss (**B**) and clinical GvHD score (**C**) were measured and are presented as mean ± SEM (2-way ANOVA). (**D**) Survival of mice was monitored and is presented as percentage survival. *n* = 5 mice per group (log-rank Mantel-Cox test). Data are shown as mean ± SEM. *P < 0.05; **P < 0.01; ***P < 0.001.

To further confirm these findings and to explore the potential clinical translation of 6PGD inhibition on GvT effects, we treated mice in the GvT model with 6AN. BALB/c mice received lethal irradiation (8.5 Gy, ^137^Cs) on day –1 and on day 0 were injected with A20-Luc^+^ tumor cells (0.1 × 10^6^ cells) 4 hours before transplantation with 3.5 × 10^6^ TCD-BM cells from C57BL/6 WT mice and 0.2 × 10^6^ splenic naive T cells from WT donors. The mice were treated with vehicle or 6AN (0.5 mg/kg, administered daily via i.p. injection), starting on day 0 until the endpoint. Examination of tumor growth showed that control mice receiving only tumor cells and TCD-BM treated with either vehicle or 6AN exhibited significant tumor growth (Figure 7A). Tumor growth was controlled in recipients of T cells from both vehicle and 6AN treatment, indicating that the T cells retained GvT activity (Figure 7A). In mice with donor T cells plus tumor cells, 6AN-treated recipients further exhibited reduced aGvHD severity compared with those receiving vehicle, as shown by higher body weight (Figure 7B), lower clinical GvHD scores (Figure 7C), and enhanced survival (Figure 7D). These results show the effect of 6PGD inhibition on donor T cells to mitigate aGvHD while maintaining the GvT effect. Next, we examined the direct effect of 6AN on tumor cells. We have previously shown that 6PGD blockade enhances the antitumor function of endogenous (autologous) T cells (15). Other investigators have shown that 6PGD function supports tumor cell growth (35, 36). We used a syngeneic GvT murine model with and without 6AN, showing that 6AN treatment reduces tumor growth, resulting in increased survival, without changes in body weight or GvHD clinical score (Supplemental Figure 11, A–C). We showed that in vitro exposure of A20 tumor cells to increased concentration of 6AN (5, 10, and 20 μM) resulted in a higher apoptotic rate (Supplemental Figure 11D) and lower expression of the Ki67 proliferation marker (Supplemental Figure 11E). These results confirm the direct additive effect of 6AN on tumor control along with the enhanced antitumor function of donor T cells, while ameliorating the aGvHD severity, highlighting the potential clinical translatability of 6AN treatment after allo-HCT.

The ability to preferentially modulate T cell responses through metabolic reprogramming at the 6PGD checkpoint provides a promising therapeutic strategy for achieving improved outcomes in allo-HCT.

## Discussion

In this study, we demonstrate that blocking 6PGD, the second rate-limiting enzyme in the PPP, significantly decreases the severity of aGvHD. Inhibiting 6PGD by selective genetic deletion in T cells or by pharmacologic inhibition by 6AN led to decreased aGvHD severity, with no impact on the GvT effect after allo-HCT.

The increase in PPP pathway activity in allogeneic T cells aligns with previous findings that demonstrate increased glucose metabolism in GvHD (5, 8). Elevated uptake of glucose analogs correlates with donor cell infiltration and GvHD severity (37). Preclinical models have confirmed the important role of glycolysis in allogeneic T cells. Glycolytic inhibition through 2DG or Glut1 deficiency in donor T cells reduces aGvHD (5, 8). In this study, the selective deletion of 6PGD in donor T cells during allo-HCT led to changes in the ratio of effector to naive T cells in *Pgd^fl/fl^Cd4^Cre^* mice compared with WT (*Pgd^fl/fl^*) mice. Therefore, naive T cells were used as the source of T cells for allo-HCT. However, in vitro and in vivo assays showed that deficiency of 6PGD in T cells caused reduced T cell proliferation, manifested by a 25%–75% reduction in in vitro CD4^+^ and CD8^+^ proliferation and 40%–60% reductions in vivo in CD4^+^ and CD8^+^ subsets, especially on days +7 and +14 (detailed in Figure 2). This emphasizes the critical dependence of allogeneic T cells on glycolysis (9, 38), particularly the PPP, to meet proliferative and cytotoxic demands.

We have further elucidated how allogeneic T cells utilize glucose through the PPP during T cell proliferation and expansion. Specifically, 6PGD is upregulated in allogeneic T cells 7 days after allo-HCT. Blocking 6PGD, either genetically or pharmacologically, significantly reduced T cell proliferation without apoptosis. Thus, 6PGD activity is essential for T cell progression through the cell cycle. Our results are consistent with earlier studies showing that PPP inhibition leads to G_2_/M cell cycle arrest in cancer cells (39). While previous research attributed this arrest to insufficient R5P levels for DNA synthesis (40), we found that R5P levels remain unaffected. In allogeneic T cells, there is compensation by the non-oxidative branch of the PPP. NADPH production is significantly reduced with PPP inhibition, leading to elevated superoxide levels that may trigger downstream signaling resulting in cell cycle arrest, reduced T cell proliferation, and diminished T cell absolute numbers. There was no significant difference in apoptosis between WT and 6PGD-deficient T cells, indicating that sufficient NADPH production via G6PD prevented excessive superoxide accumulation that could cause cell death. We also observed that 6PGD blockade induces metabolic reprogramming in allogeneic T cells, shifting their metabolism from glycolysis to OXPHOS, predominantly utilized by naive and resting T cells. However, activated T cells exhibit an increase in OXPHOS alongside an upregulation of glycolysis (41–43). This metabolic shift following 6PGD blockade is likely a result of PPP inhibition: redirecting glucose into the non-oxidative PPP and OXPHOS pathways. Additionally, most T cells remain in a naive state after 6PGD blockade, relying primarily on OXPHOS for energy. This metabolic switch after PPP inhibition represents an advantage over strategies that disrupt T cell metabolism or that are toxic for T cells such as targeting glycolysis (32).

Since allo-HCT is employed to treat patients with hematologic malignancies, it was critical to understand how PPP inhibition affected the GvT effect. 6PGD-deficient T cells during allo-HCT retained their ability to improve recipient survival by inhibiting tumor growth without inducing severe aGvHD. Previous studies found that CD8^+^ T cell cytotoxic granule expression (e.g., granzyme B and perforin) is not regulated by glycolysis, contributing to a preserved GvT effect (8). We demonstrated in vitro that 6PGD-deficient T cells generated improved tumor killing, with increased granzyme B expression. Although granzyme B has been associated with GvHD, the absolute number of effector T cells in 6PGD-deficient mice was lower than in the WT group. This suggests that there may not be sufficient granzyme B–positive T cells to induce severe GvHD (44), but their numbers are adequate to maintain a GvT effect. Further studies are needed to evaluate the impact of 6AN on in vitro and in vivo cytotoxic T cell generation and function, by assessing the expression of cytotoxic molecules including Fas/FasL, granzymes A and B, perforin, and proinflammatory cytokines such as IFN-γ and TNF-α. Additionally, it is crucial to investigate the cytolytic capacity of these cytotoxic T cells to determine whether 6AN affects their functional cytotoxicity. Clinical studies have proposed that the allogeneic T cell dose required to induce aGvHD is approximately 10-fold higher than the dose needed to achieve a GvT effect; however, this is dependent on the different T cell subsets (45). Thus, a small population of 6PGD-deficient T cells appears to be sufficient to control tumor progression without triggering severe aGvHD.

To evaluate the translational potential of targeting 6PGD, we used 6AN, a selective 6PGD inhibitor, in murine and xenogeneic allo-HCT models. 6AN significantly reduced aGvHD severity, mirroring the phenotype observed with 6PGD-deficient T cells. These findings underscore the translational value of targeting the PPP to ameliorate GvHD severity and mortality. 6AN was used in this study but had been found to have minimal antitumor effects at lower doses and toxicity when dose-escalated in patients with advanced cancers (46). Development of novel and less toxic 6PGD inhibitors will be critical for further evaluation to minimize GvHD while preserving the GvT effect.

In summary, using conditional 6PGD-deficient murine models and a selective 6PGD inhibitor, we demonstrate that the PPP is critical for alloreactive T cell proliferation and cell cycle progression during aGvHD. Targeting 6PGD effectively mitigates aGvHD while preserving GvT activity. Future efforts should focus on testing selective 6PGD inhibitors to optimize clinical outcomes in allo-HCT. Our study establishes the PPP as a viable target for preventing aGvHD without compromising GvT efficacy.

## Methods

### Sex as a biological variable

Both male and female mice were used in all the in vivo studies.

### Mice and A20-Luc+ cell line

The mouse strains C57BL/6J (B6, H-2^b^, CD45.2^+^), B6.SJL-Ptprc^a^ Pepc^b^/BoyJ (B6, H-2^b^, CD45.1^+^), B6.Cg-Tg1Cwi/BfluJ (*Cd4^Cre^*), C3.SW-H2b/SnJ (C3H.H2^b^), BALB/c (H-2^d^), and NOD.Cg-*Prkdc^scid^ Il2rg^tm1Wjl^* Tg(HLA-A/H2-D/B2M)1Dvs/SzJ (NSG) were purchased from The Jackson Laboratory. *Pgd^fl/fl^* mice were provided by Pankaj Seth (Beth Israel Deaconess Medical Center, Boston, Massachusetts, USA) and crossed with *Cd4^Cre^* mice to generate *Pgd^fl/fl^Cd4^Cre^* mice. Female mice aged 6 to 12 weeks were age matched across different groups. All mice were housed in specific pathogen–free (SPF) facilities at the University of Minnesota and the Roswell Park Comparative Oncology Shared Resources (COSR). The A20-Luc^+^ cell line was provided by Xuefang Cao (Department of Microbiology and Immunology, University of Maryland School of Medicine, Baltimore, Maryland, USA) and cultured in RPMI 1640 (with L-glutamine and 2000 mg/L D-glucose, Corning) supplemented with 10% fetal bovine serum (FBS; Gibco), and 1% penicillin/streptomycin (MilliporeSigma).

### Murine T cell isolation and culture

Murine CD4^+^ and CD8^+^ T cells were purified from spleens and lymph nodes by negative selection as previously described (47). Pan naive T cells were isolated using EasySep Mouse Pan-Naive T Cell Isolation Kit (STEMCELL Technologies). Isolated T cells were cultured in RPMI 1640 (with L-glutamine and 2000 mg/L D-glucose, Corning) supplemented with 100 U/mL recombinant human IL-2 (rhIL-2, BioLegend), 10% FBS (Gibco), 1 mM HEPES (MilliporeSigma), 2 mM GlutaMax (Thermo Fisher Scientific), 1 mM sodium pyruvate (Thermo Fisher Scientific), 1× penicillin/streptomycin (MilliporeSigma), 50 μg/mL gentamicin (gentamicin sulfate, liquid, Corning), and 55 mM 2-mercaptoethanol (MilliporeSigma). For stimulated cultures, the culture plate was coated with 10 μg/mL functional-grade anti-CD3ε (catalog 14-0031-82, eBioscience) and 10 μg/mL functional-grade anti-CD28 (catalog 14-0281-82, eBioscience) and washed with PBS prior to seeding.

### Flow cytometry analysis

The following procedure was used for murine cell isolation: Spleens were harvested, mechanically dissociated, passed through a 70 μm pore cell strainer, and the RBCs were lysed by ammonium-chloride-potassium (ACK) lysis buffer according to manufacturer’s protocol (Thermo Fisher Scientific). Livers were harvested, mechanically dissociated, passed through a 70 μm pore cell strainer, resuspended in 40% Percoll PLUS (Catalog 17544502, Cytiva) density gradient media, and centrifuged at 600*g* for 20 minutes. The cell pellet was collected, and RBCs were lysed by ACK lysis buffer. For in vitro cultured cells, T cells were harvested on day 4 after activation. The harvested cells were suspended in FACS buffer (1% FBS in PBS) and stained for surface markers for 30 minutes at 4°C. An Aqua LIVE/DEAD Fixable Dead Cell Stain dye (Thermo Fisher Scientific) was used to separate live and dead cells. For cell cycle dye staining, Vybrant DyeCycle Violet Stain (catalog V35003, Thermo Fisher Scientific) was used, according to manufacturer’s instruction.Intracellular cytokine staining was performed using a fixation/permeabilization kit (BD Biosciences). For cytokine staining, cells were stimulated in vitro with phorbol myristate acetate (50 ng/mL; Sigma-Aldrich) and ionomycin (1 μg/mL; Sigma-Aldrich) in the presence of GolgiPlug (BD Biosciences) for 4 hours prior to staining. In our studies, BUV395-CD8a (catalog 563786, BD Biosciences), BUV661-CD122 (catalog 741493, BD Biosciences), BUV805-CCR7 (catalog 742065, BD Biosciences), Brilliant Violet 421–CXCR3 (catalog 155907, BioLegend), BV605-CD45RA (catalog 740350, BD Biosciences), FITC–TNF-α (catalog 506304, BioLegend), NovaFluor Blue 610-70S–CD44 (catalog M010T02B06, eBioscience), PerCp-Cy5.5–H2K^b^ (catalog 116516, BioLegend), PE–GM-CSF (catalog 505406, BioLegend), PE/Dazzle 594–Sca-1 (catalog 108138, BioLegend), Alexa Fluor 647–IFN-γ (catalog 557735, BD Biosciences), and Alexa Fluor 700–CD62L (catalog 104426, BioLegend) were used.

### aGvHD models

#### Fully allogeneic model.

BALB/c (H-2^d^, The Jackson Laboratory) mice were lethally irradiated (8.5 Gy;^137^Cs source, single fraction, day –1) and given i.v. 3.5 × 10^6^ TCD-BM cells from C57BL/6 (H-2^b^, The Jackson Laboratory) with or without 0.2 × 10^6^ purified naive splenic pan-T cells from fully allogeneic donor *Pgd^fl/fl^* or *Pgd^fl/fl^Cd4^Cre^* C57BL/6 mice (15). *Pgd^fl/fl^* mice were provided by Pankaj Seth (Beth Israel Deaconess Medical Center, Boston Massachusetts, USA) through TWMF. 6PGD conditional KO mice were generated by crossing *Pgd^fl/fl^* mice with *Cd4^Cre^* mice (The Jackson Laboratory) (15), generating *Pgd^fl/fl^Cd4^Cre^* transgenic mice.

#### Minor histocompatibility antigen disparate model.

C3H/SW mice (H-2^b^, The Jackson Laboratory) were lethally irradiated (11 Gy; ^137^Cs source, single fraction, day –1) and transplanted i.v. with 3.5 × 10^6^ TCD-BM cells with or without 0.5 × 10^6^ splenic naive T cells from *Pgd^fl/fl^* or *Pgd^fl/fl^Cd4^Cre^* C57BL/6 mice on day 0. In all GvHD models, monitored survival is reported as percentage survival. GvHD clinical scores based on posture, motility, hair loss, skin integrity, and weight loss were monitored twice weekly (24, 25). The primary experimental endpoint was survival, with mice monitored daily for clinical signs of disease progression. Mice were euthanized upon reaching a moribund state, defined as unresponsiveness to gentle stimulation, in accordance with institutional animal care guidelines. Additionally, body weight was monitored twice a week and served as another indicator of disease severity and general health status. These criteria allowed consistent assessment of treatment outcomes.

### Pharmacological blockade of 6PGD

Pharmacological blockade of 6PGD was tested in lethally irradiated (6 Gy; x-ray source, day –1) BALB/c mice transplanted i.v. with 1 × 10^7^ fully allogeneic BM cells with or without 1 × 10^6^ splenic T cells from WT C57BL/6 mice on day 0 (48). Mice were i.p. injected daily with 0.5 mg/kg 6AN (MilliporeSigma) or vehicle (DMSO) from day 0 until day 60. For xenogeneic GVHD studies, NSG mice were sublethally irradiated with a single fraction of 2.5 Gy (^137^Cs source) on day –1 and transplanted i.v. with 2 × 10^6^ human PBMCs on day 0. Fresh deidentified PBMCs were provided by the Data Bank and BioRepository (DBBR) at Roswell Park Comprehensive Cancer Center. In xenogeneic murine models, 6AN (1 mg/kg; MilliporeSigma) or vehicle (DMSO) was administered i.p. every 2 days.

### GvT model

BALB/c mice were lethally irradiated (8.5 Gy, ^137^Cs source, day –1). On day 0, hosts were injected i.v. with 3.5 × 10^6^ TCD-BM cells with or without 0.2 × 10^6^ purified naive splenic pan-T cells (Negative Selection Isolation Kit, STEMCELL Technologies) from *Pgd^fl/fl^* or *Pgd^fl/fl^Cd4^Cre^* C57BL/6 mice. On day 0, experimental cohorts were also injected i.v. with 0.1 × 10^6^ A20-Luc^+^ (BALB/c) tumor cells. Tumor burden was measured by BLI weekly (24, 25). Body weight loss, clinical GvHD score, and survival were monitored daily.

These experiments were conducted in 2 different centers (Roswell Park and the University of Minnesota) and included 2 different aGvHD and GvT models. These settings strengthen the results and confirm the role of 6PGD inhibition in allo-HCT outcomes independent of transplantation model.

### scRNA-seq

To examine the metabolic changes in allogeneic T cells, allo-HCT in the fully allogeneic model was conducted as described above. Splenic donor T cells (CD45^+^H2K^b+^H2K^d–^TCRβ^+^) were harvested, sorted on days +7 and +14, and examined for T cell clusters using scRNA-seq. Sorted cells were analyzed with AOPI stain (Revvity Health Sciences) and a Cellometer K2 automated cell counter (Nexcelom) to assess concentration, viability, and to confirm the absence of clumping and debris. scRNA-seq was performed as previously described (49). Bioinformatic analyses were conducted using the Seurat (v5.0.0) R package for scRNA-seq (50). Oxidative PPP gene signatures were examined within T cell clusters.

scRNA-seq analysis workflow: For the 10× Genomics Chromium libraries, the raw sequencing data were processed using Cellranger software (Cell Ranger Single-Cell Software Suite) (http://software.10xgenomics.com/single-cell/overview/welcome). Then, the filtered gene-barcode matrices, which contain barcodes with the unique molecular identifier (UMI) count that passed the cell detection algorithm, were used for further analysis. Doublet detection was performed using Scrublet (51) for each sample, and the cells with a doublet score higher than the predicted threshold were excluded in the analysis. All the downstream analyses were performed using the Seurat single-cell data analysis R package (50). First, cells with low RNA content and with a low number of detected genes (<500), very high number of detected genes (>7500) due to doublets, or higher mitochondrial RNA content (>15%) were filtered out from the analysis. Then, the normalized and scaled UMI counts were calculated using the SCTransform method in Seurat. Dimension reductions including principal component analysis (PCA), uniform manifold approximation and projection (UMAP), and t-SNE were carried out using highly variable genes. Data clustering was identified using shared nearest neighbor–based (SNN-based) clustering on the first 10–30 principal components. Subsequently, the cell clusters can be annotated by the known gene markers or by identifying differentially expressed genes for each cluster. Gene set enrichment analysis (GSEA) was done using the fgsea (52) R package with average log_2_(fold change)calculated with FindMarkers from Seurat. The hallmark and canonical pathways from the Molecular Signatures Database (MSigDB; https://www.gsea-msigdb.org/gsea/msigdb) were used in GSEA analysis.

### Co-adaptive transfer during allo-HCT

To examine the proliferative capacity of 6PGD-deficient T cells during allo-HCT, naive T cells were isolated from CD45.1^+^ WT and CD45.2^+^ *Pgd^fl/fl^CD4^Cre^* mice, mixed at a 1:1 ratio, and labeled with CFSE (Thermo Fisher Scientific) for 30 minutes at 37°C. On day 0, 3.5 × 10^6^ TCD-BM cells from C57BL/6 and 0.4 × 10^6^ mixed CFSE-labeled T cells were injected i.v. into lethally irradiated (8.5 Gy; ^137^Cs source) BALB/c mice. Proliferation was determined on day +3 by CFSE dilution in splenic CD45.1^+^ and CD45.2^+^ T cells. The frequencies of CD45.1^+^ WT and CD45.2^+^ *Pgd^fl/fl^Cd4^Cre^* T cells in spleen, mesenteric lymph nodes, and liver were examined on day +7 after transplantation. Target tissues were collected, mechanically dissociated, passed through a 70 μm cell strainer, RBCs were lysed with ACK lysis buffer according to the manufacturer’s protocol (Thermo Fisher Scientific), and remaining cells examined by flow cytometry.

### Gene expression by qPCR

To confirm the screening scRNA-seq data, WT BALB/c mice were irradiated (8.5 Gy, ^137^Cs source) and transplanted i.v. with 3.5 × 10^6^ TCD-BM cells (isolated from C57BL/6 mice) with or without 0.2 × 10^6^ splenic naive C56BL/6 T cells. Alloreactive T cells (CD45^+^H2K^b+^H2K^d–^TCRβ^+^) were sorted using a SONY MA900 sorter on day +7 after allo-HCT. The expression of the *Pgd* gene (6PGD) was examined in sorted T cells on day +7 compared to day 0 naive T cells by real-time quantitative PCR using the ABI 7300 Real-Time PCR system (Applied Biosystems). Total RNA was extracted with RNeasy Mini Kit (QIAGEN). Total DNA-free RNA was used for mRNA isolation and library construction. The expression levels of *G6pdx* (TaqMan probe: Mm04260097_m1), *Pgls* (TaqMan probe: Mm00452601_m1), and *Pgd* (TaqMan probe: Mm01263703_m1) were measured using the TaqMan RNA-to-CT 1-Step Kit (Thermo Fisher Scientific). The gene expression was normalized to18S rRNA housekeeping gene expression as ΔCt = Ct (target gene) – Ct (18S rRNA). Changes between day 0 and day +7 were calculated as fold change = 2^ΔΔCt^, where ΔΔCt = Ct (target gene) – Ct (target gene at baseline).

### NADPH measurement

NADP^+^/NADPH levels were measured by a colorimetric assay according to the manufacturer’s instructions (Abcam). Briefly, T cells were cultured for 72 hours with vehicle or 6AN. The cells were lysed, incubated with the reaction mixture, followed by monitoring the absorbance at 450 nm, which is proportional to NADPH concentration.

### SCENITH

Vehicle- or 6AN-treated T cells were analyzed according to the standard procedure described in the original publication (31). Briefly, activated T cells treated with vehicle or 6AN for 72 hours were harvested from culture plates, washed, and incubated with metabolic inhibitors (100 mM 2DG, 1 μM oligomycin, and a combination of 100 mM 2DG and 1 μM oligomycin) in complete RPMI 1640 for 15 minutes. Puromycin (10 μg/mL) was added to the media containing 6AN, and the cells were incubated for an additional 30 minutes. After washing, the cells were stained for surface markers and puromycin. Translational changes during metabolic inhibition were determined by calculating the geometric mean fluorescence intensity (gMFI) of puromycin and analyzed for glucose and mitochondrial dependence.

### Seahorse bioenergetics analysis

Splenic naive T cells were isolated from *Pgd^fl/fl^* or *Pgd^fl/fl^Cd4^Cre^* mice and activated with plate-bound anti-CD3 and anti-CD28 (10 μg/mL each) plus rmIL-2 (100 ng/mL) for 4 days. In another set, naive T cells were stimulated in presence of 6AN (10 μM) or vehicle control. On day 4, the harvested cells were examined in a XFe96 Extracellular Flux Analyzer (Seahorse Bioscience) for glycolysis function by examination of the extracellular acidification rate (ECAR; mpH/min), and for the mitochondrial function by examining the mitochondrial oxygen consumption rate (OCR; O_2_ pmol/min). For this, 2 × 10^5^ cells/well were seeded on Cell-Tak-coated Seahorse XFe96 (Agilent) culture plates in assay media. The ECAR was analyzed in 4 consecutive stages: basal, glycolysis induction (by adding 10 mM glucose), maximal glycolysis (by adding 2 mM oligomycin), and inhibition of glycolysis (by adding 100 mM 2DG). The OCR was measured in the following stages: basal respiration, inhibition of complex V (by adding 2 mM oligomycin), maximal respiration (by adding 1 mM carbonyl cyanide 4-(trifluoromethoxy) phenylhydrazone [FCCP]), and inhibition of the electron transport chain (by adding 1 mM rotenone plus 1 mM antimycin A).

### Mitochondrial mass and mitochondrial potential analysis

Mitochondrial phenotype also was examined by flow cytometry. For this, splenic naive T cells from *Pgd^fl/fl^* or *Pgd^fl/fl^Cd4^Cre^* mice were activated in vitro by plate-bound anti-CD3 and anti-CD28 (10 μg/mL each) plus rmIL-2 (100 ng/mL) for 4 days. Harvested cells were stained with 200 nM MitoTracker Deep Red FM (Thermo Fisher Scientific) for mitochondrial mass and 200 nM tetramethylrhodamine ester (TMRE) (Thermo Fisher Scientific) for mitochondrial membrane potential for 30 minutes at 37°C. The stained cells were analyzed by the cells were analyzed using the BD LSRFortessa cell analyzer (BD Biosciences).

### Transmission electron microscopy

Splenic naive T cells were isolated from *Pgd^fl/fl^* or *Pgd^fl/fl^Cd4^Cre^* mice and activated with anti-CD3 and anti-CD28 (10 μg/mL each) plus rmIL-2 (100 ng/mL) for 4 days. The harvested T cells on day 4 were fixed in 4% glutaraldehyde and stained for electron microscopy. Ultrathin section cuts of fixed cells were placed on a copper grid, stained with lead citrate, and examined in a JEOL 1200EX transmission electron microscope or a Tecnai G2 Spirit BioTWIN.

### SIRM analysis

To investigate glucose consumption following the 6PGD blockade, T cells were activated in the presence of 6AN (10 μM) or vehicle control. On day 4, T cells were harvested and treated with tracing media consisting of glucose-free RPMI 1640 media enriched with 10% dialyzed FBS (Life Technologies), 20 mM HEPES, 0.05 mM 2-mercaptoethanol, and 1% penicillin-streptomycin. This medium also included the stable isotope–enriched nutrient D_7_-D-glucose (10 mM; Cambridge Isotope Laboratories). Cells were collected at the 8-hour time point, and the metabolite concentrations were determined via ion chromatography-ultra high-resolution mass spectrometry (IC-UHR-MS) using previously established methods (15). The concentration of each metabolite was computed and normalized to total protein abundance (μmole/g protein).

### Western blotting

For Western blot analyses, T cells (CD45^+^H2K^b+^H2K^d–^TCRβ^+^) were sorted using a SONY MA900 sorter on day +7 after allo-HCT and lysed in radioimmunoprecipitation assay (RIPA) buffer (Thermo Fisher Scientific). The original T cells injected on day 0 served as baseline control. The RIPA buffer was supplemented with 1% protease and phosphatase inhibitor (Thermo Fisher Scientific) according to the manufacturer’s instructions. Samples were run in SDS-PAGE (Bio-Rad) to separate the target proteins transferred to a PVDF membrane (Bio-Rad). The membranes were blocked with EveryBlot Blocking Buffer (Bio-Rad) and treated with primary antibodies of rabbit anti-PGD (catalog 14718-1-AP, Proteintech) and rabbit anti-Vinculin (catalog 13901, Cell Signaling Technology) at 1:1000 dilution and then HRP-conjugated anti-rabbit IgG secondary antibody (catalog 7074, Cell Signaling Technology) at 1:3000 dilution. The membranes were developed with Clarity Max Western ECL Substrate (Bio-Rad). The images were captured using an Odyssey Fc (LI-COR).

### Statistics

Statistical evaluations were conducted using Prism 10 (GraphPad). Group differences were assessed using the 2-tailed Student’s *t* test for comparisons between 2 groups and 1-way ANOVA for comparisons involving more than 2 groups, followed by Tukey’s post hoc test. Tumor growth was analyzed using 2-way ANOVA with Tukey’s post hoc analysis. A *P* value of less than 0.05 was considered indicative of statistical significance between groups. Data presented in the figures are expressed as mean ± SEM.

#### Study approval.

Animal studies were conducted in accordance with a protocol reviewed and approved by the Institutional Animal Care and Use Committee (IACUC) of the University of Minnesota (2403-41944A) and Roswell Park (1143M). *Pgd^fl/fl^* or *Pgd^fl/fl^Cd4^Cre^* C57BL/6 mice were bred and maintained in Roswell Park animal facility under IACUC protocol no. 1140.

### Data availability

Values for all data points found in graphs are in the Supporting Data Values file. The scRNA-seq data can be accessed through the NCBI Gene Expression Omnibus (GEO GSE289995).

## Author contributions

Experimental design and execution were conducted by SD, EK, BRB, and HM. Data interpretation and analysis was performed by SD, EK, PLM, BRB, and HM. QY, JEC, SH, and KLH helped with in vitro and in vivo experiments. Histopathology evaluations were done by JQ. Bioinformatical and scRNA-seq transcriptomics analysis were done by PKS and JW. Tracing studies were performed by SD, RMH, ANL, and TWMF. Xenogeneic GVHD studies were performed by SH and KLH. The manuscript was written and edited by SD, EK, PLM, BRB, and HM.

## Funding support

This work is the result of NIH funding, in whole or in part, and is subject to the NIH Public Access Policy. Through acceptance of this federal funding, the NIH has been given a right to make the work publicly available in PubMed Central.

- NIH grant R00 HL155792 (to HM).
- V Foundation (to HM).
- Roswell Park Alliance Foundation (to HM).
- American Society of Hematology Graduate Hematology Award (to EK).
- NIH grants R37AI34495, R01HL155114, and P01HL158505 (to BRB).
- Brendan and Elise McCarthy (to PLM).
- National Cancer Institute grant P30CA016056.
- NIH grant P30CA177558 (to BM Evers, Metabolism Shared Resources at the University of Kentucky); partial support for IC-UHR-MS data acquisition.

## Supplementary Material

Supplemental data

Unedited blot and gel images

Supporting data values

## Version 1 12/08/2025

Electronic publication
