# Supplementary material for: Targeting the pentose phosphate pathway mitigates graft-versus-host disease by rewiring alloreactive T cell metabolism
Source: JCI Insight. 2025 Dec 8;10(23):e192774. doi: 10.1172/jci.insight.192774 (PMC12890487; doi:10.1172/jci.insight.192774)
Supplement: Supplemental data [file jciinsight-10-192774-s151.pdf]

Supplemental Figures:

A

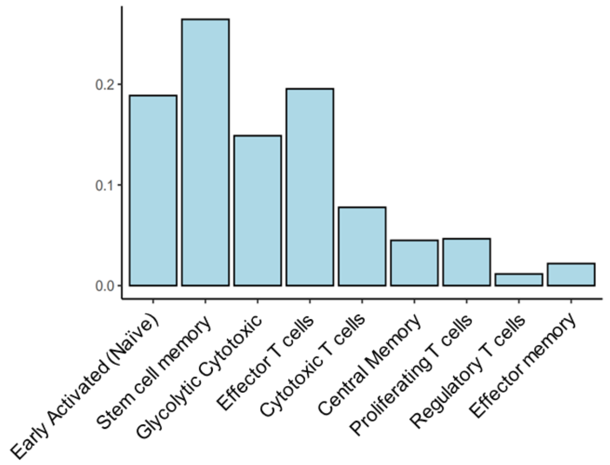

B

Cluster name	Signature genes
Early Activated (Naïve)	S100A4, S100A6, Lgals1, Crip1, Serpinb6b
Stem cell memory	Mki67, Stmn1, Top2a, Tuba1b, Cdk1, Pclaf, Tubb5, Pcan
Glycolytic Cytotoxic	Ldha, Gapdh, Tpi1, pkm, Pfn1, Gzmk, Ccnb2, Cenpa ,Cdc20
Effector T cells	CD7, Ypel3, Ccl5, Malat1, Btg1, Nkg7, Cxcr6, Hcst
Cytotoxic T cells	Gzma, Gzmb, Gzmk, Gzmk, Ccr2, Klrd1, Ly6c2
Central Memory	Iftm1, Iftm2, Iftm3, IL7r, Anxa1
Proliferating T cells	Zfp36l1, Tox, Havcr2, Samsn1, Ifng
Regulatory T cells	Foxp3, Il10, Gzmb, Gimap6, Ctla4, Maf, Ikzf2, Itgae, Socs2, Capg
Effector Memory	mt-Co1, mt-Co2, mt-Co3, mt-Atp6, mt-Nd4, Mt-Nd5, Lars2

C

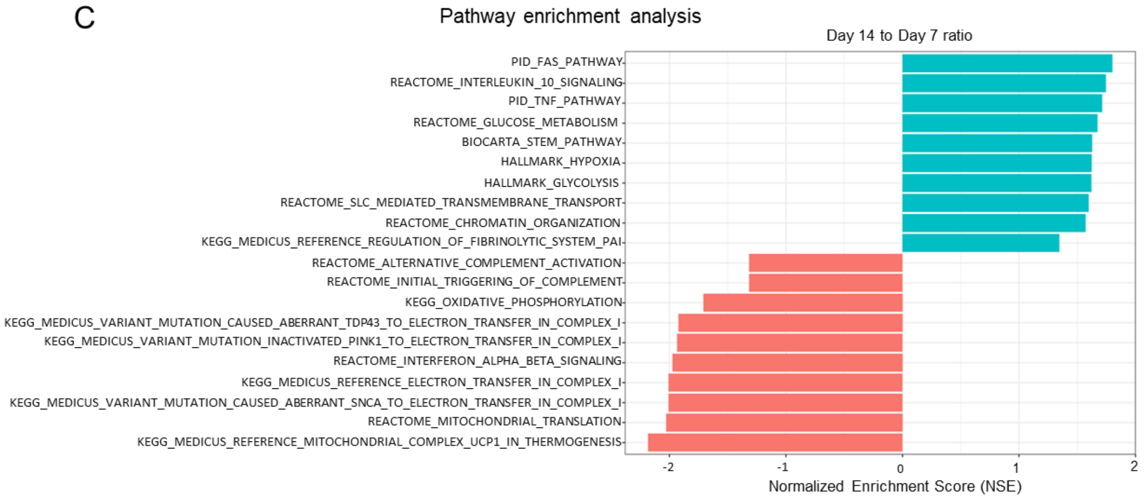

Figure S1: T cells frequencies examined in single cell RNA sequencing (scRNA seq).

(A-B) BALB/c (H-2^d) mice were lethally irradiated (8.5 Gy) on day -1 and transplanted with 3.5×10^6 TCD-BM with or without 0.2×10^6 splenic T cells from WT C57BL/6 (H-2^b) mice on day 0. Splenic donor T cells (CD45+, H2k^{b+}, H2k^{d-}, TCRβ^+) were sorted on day +7 and day +14, examined for T cell clusters using scRNA seq and combined for analysis. The percentage of T cells in each cluster (A) and the signature genes for each cluster (B) is shown. The T cell clusters were defined based on previously published references (17-22). The pathway enrichment analysis was done comparing day +14 to day +7 and top 10 upregulated and downregulated pathways are demonstrated (C).

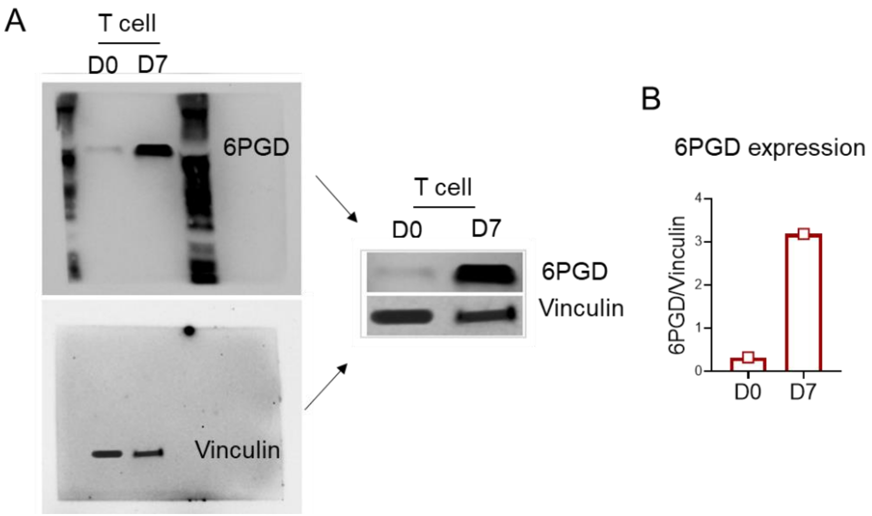

Figure S2: 6PGD expression is enhanced in T cells during GvHD development.

(A-B) BALB/c (H-2^d) mice were lethally irradiated (8.5 Gy) on day -1 and transplanted intravenously with $3.5x10^6$ TCD-BM with or without $0.2x10^6$ splenic naïve T cells from WT C57BL/6 (H-2^b) mice on day 0. Expression of 6PGD was evaluated in donor T cells at the time of transplant (D0) compared to T cells isolated from recipient spleens on day +7 (D7) post transplantation by western blot analysis. Vinculin expression served as the control. Expression levels of 6PGD were calculated based on vinculin band expression. T cells were isolated and pooled for analysis from 5 spleens. The results are representative of two independent experiments.

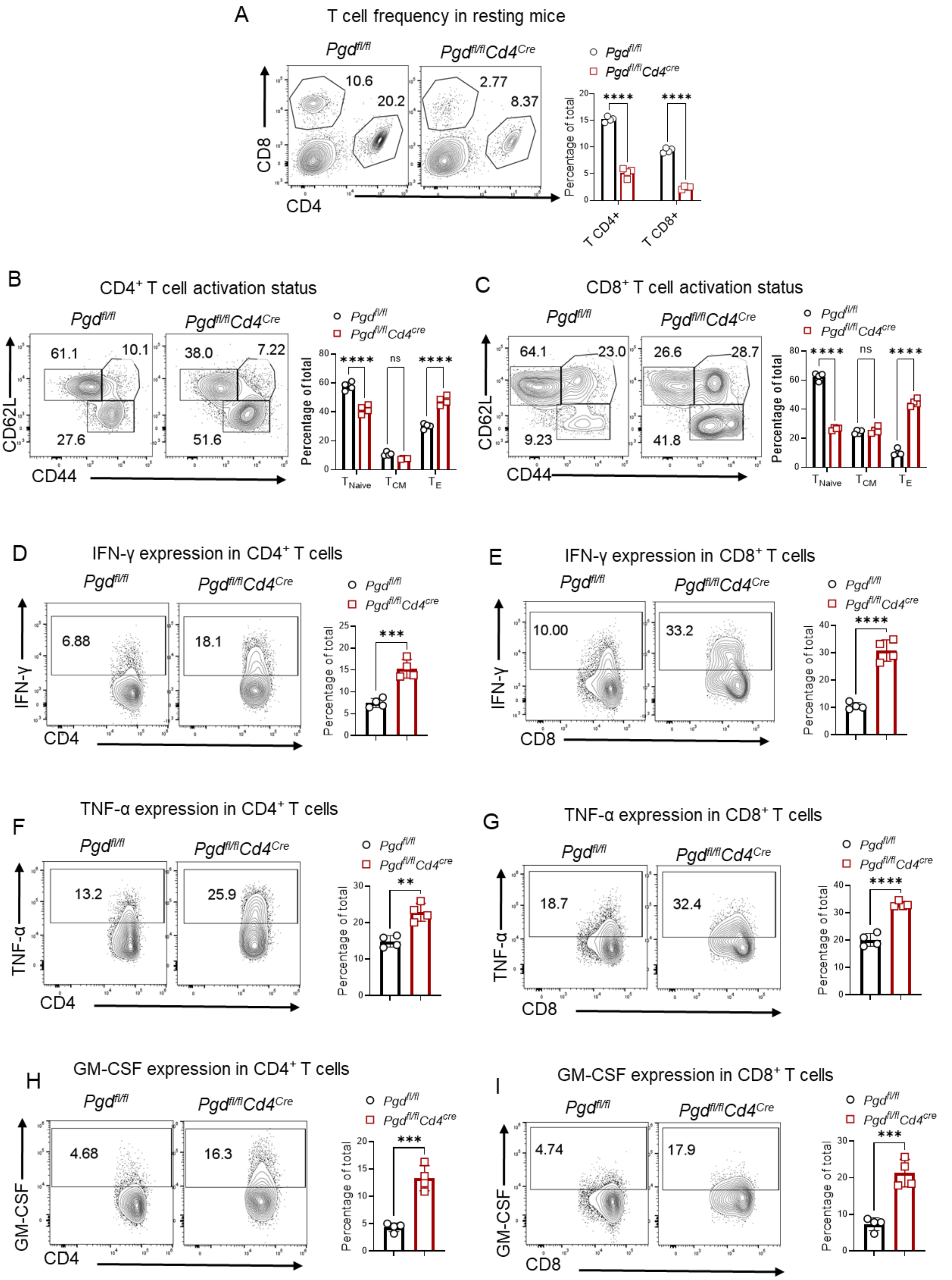

A
T cell frequency in resting mice
B
CD4+ T cell activation status
C
CD8+ T cell activation status
D
IFN-γ expression in CD4+ T cells
E
IFN-γ expression in CD8+ T cells
F
TNF-α expression in CD4+ T cells
G
TNF-α expression in CD8+ T cells
H
GM-CSF expression in CD4+ T cells
I
GM-CSF expression in CD8+ T cells
Pgdfl/fl
Pgdfl/flCd4Cre
Percentage of total

Figure S3: Blockade of 6PGD in T cells of resting mice (no antigen exposure) prevents T cell expansion while increasing effector status differentiation.

(A) To examine the intrinsic effect of 6PGD blockade on T cell phenotype, T cells from the spleens of $Pgd^{fl/fl}$ or $Pgd^{fl/fl}Cd4^{Cre}$ C57BL/6 mice in the resting state (no antigen exposure) were examined by flowcytometry. $Pgd^{fl/fl}Cd4^{Cre}$ mice had lower number of $CD4^+$ and $CD8^+$ T cells. N = 4 mice per group. The Student's t test was used to analyze statistical significance between 2 groups. The results are representative of two independent experiments.

(B-C) T cells from the mice in A were examined for differentiation status. There was a lower frequency of naïve T cells (T_{Naive}) and a higher frequency of T effector cells (T_E) in $CD4^+$ (B) and $CD8^+$ (C) T populations. N = 4 mice per group. The Student's t test was used to analyze statistical significance between 2 groups.

(D-E) T cells from the mice as in A were examined for IFN-γ production by flowcytometry. There was higher IFN-γ production by $CD4^+$ (D) and $CD8^+$ (E) T cells from $Pgd^{fl/fl}Cd4^{Cre}$ mice compared to $Pgd^{fl/fl}$ controls. N = 4 mice per group. The Student's t test was used to analyze statistical significance between 2 groups.

(F-G) T cells from the mice as in A were examined for TNF-α production by flowcytometry. There was higher TNF-α production by $CD4^+$ (F) and $CD8^+$ (G) T cells from $Pgd^{fl/fl}Cd4^{Cre}$ mice compared to $Pgd^{fl/fl}$ controls . N = 4 mice per group. The Student's t test was used to analyze statistical significance between 2 groups.

(H-I) T cells from the mice as in A were examined for GM-CSF production by flowcytometry. There was higher GM-CSF production by $CD4^+$ (H) and $CD8^+$ (I) T cells from $Pgd^{fl/fl}Cd4^{Cre}$ mice compared to $Pgd^{fl/fl}$ controls. N = 4 mice per group. The Student's t test was used to analyze statistical significance between 2 groups.

Data are shown as Mean ± SEM. $^{*}p < 0.05$; $^{**}p < 0.01$; $^{***}p < 0.001$; $^{****}p < 0.0001$.

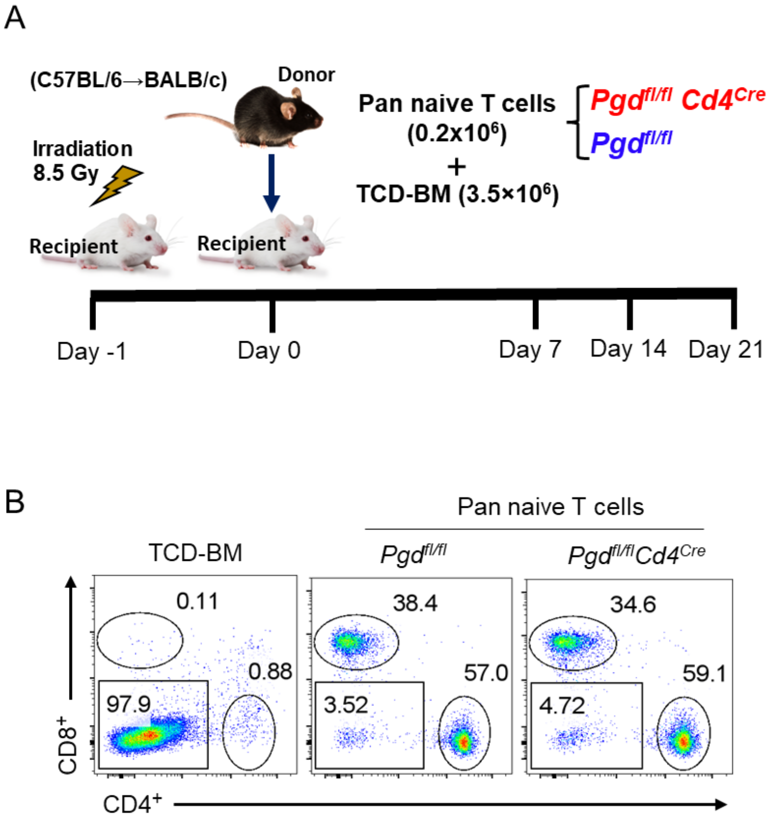

Figure S4: Purity of isolated naïve T cells used for GvHD induction.

(A-B) To evaluate aGvHD, BALB/c (H-2^d) mice were lethally irradiated (8.5 Gy) on day -1 and transplanted with 3.5×10^6 TCD-BM with or without 0.2×10^6 splenic naïve T cells from WT C57BL/6 (H-2^b) mice on day 0 (A). The purity of sorted T cells was determined by flowcytometry before injection (B). TCD-BM: T cell depleted bone marrow. Data representative of four independent repeats.

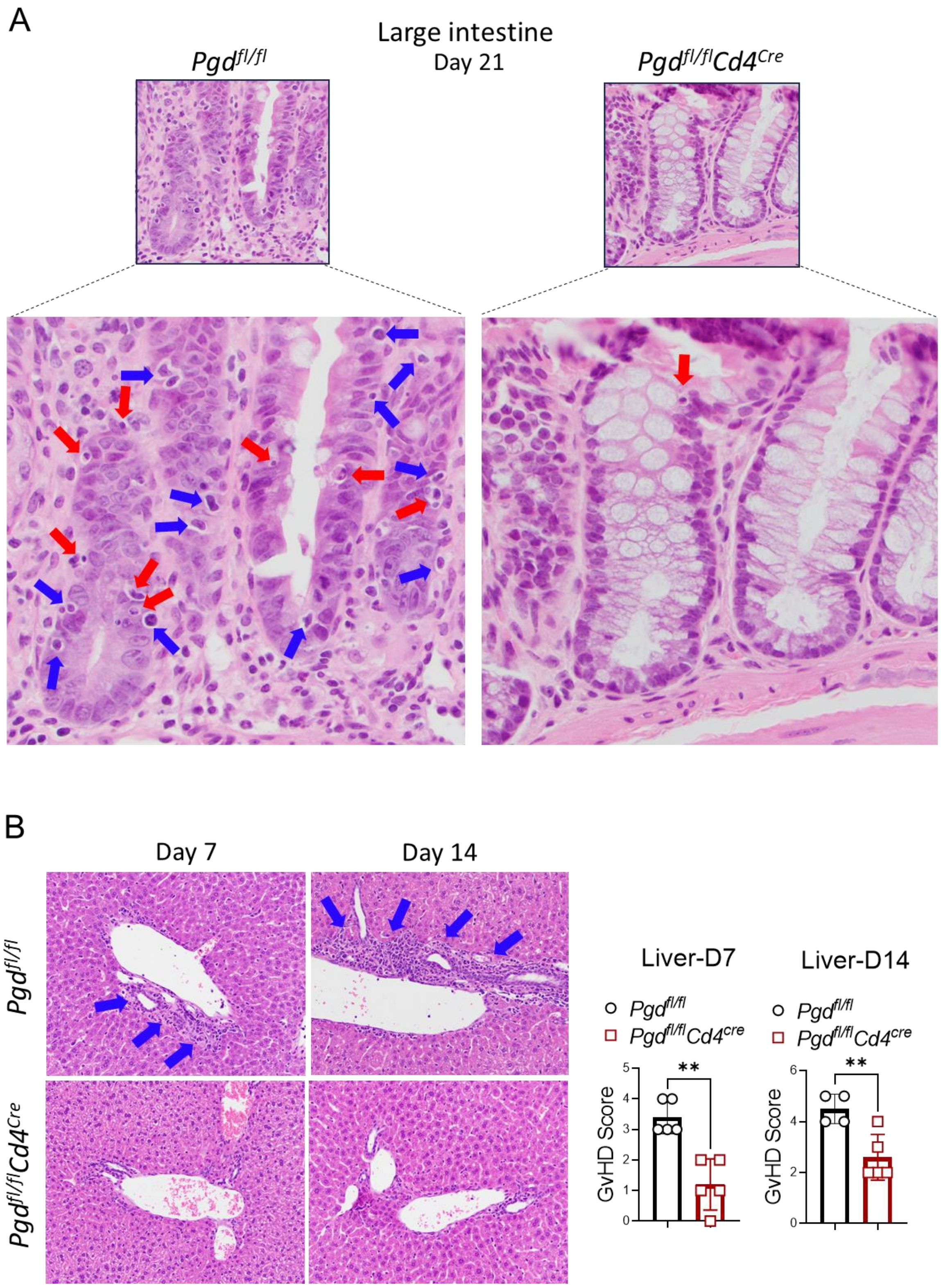

Figure S5: Histopathology of large intestine and liver after allogeneic HCT with T cells from either WT (Pgd$^{fl/fl}$) or 6PGD-deficient (Pgd$^{fl/fl}$CD4^{Cre}) donors

(A-B) Allo-HCT was conducted as in Figure 1E-G. On day +21, representative H&E-stained images of the large intestine show increased infiltration of intraepithelial lymphocytes (blue arrows) and apoptosis (red arrows) in recipients of *Pgd$^{fl/fl}$* T cells (A). Images are presented at 200X magnification (top) and at 4X enlargement (bottom). Day +7 and +14, representative H&E-stained liver images show lymphocyte infiltration (blue arrows) (B). Histological evaluations were done in a blinded manner using a previously

reported, semiquantitative scoring system[24, 25]. N = 4-5 mice per group. Statistical significance between the two groups was analyzed using Student's t test.

Data are shown as Mean ± SEM. **$p < 0.01$;

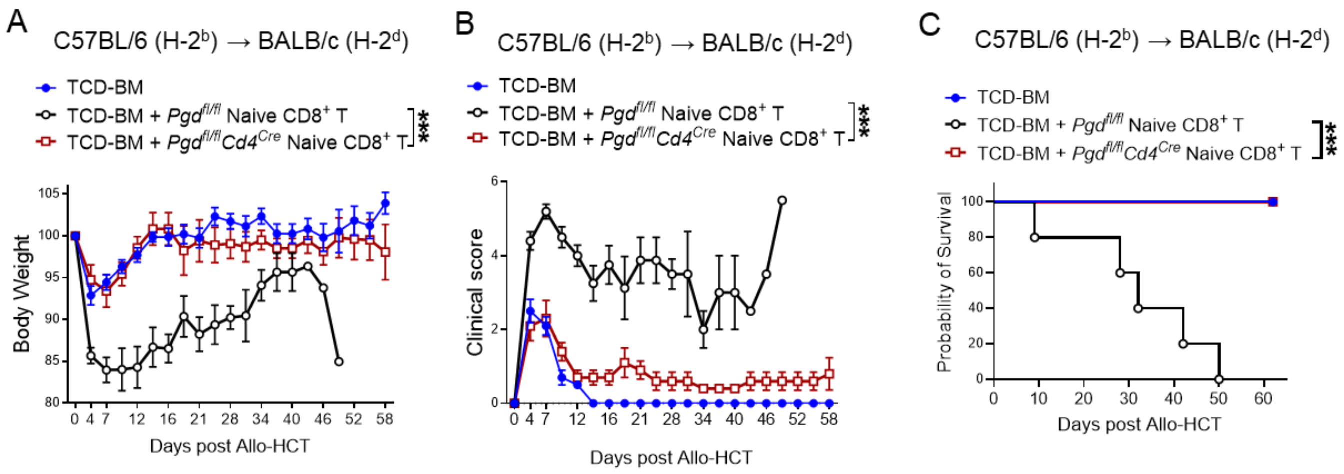

Figure S6: Blockade of 6PGD in CD8⁺ T cell ameliorates the severity of and the mortality of aGvHD.

(A-C) BALB/c (H-2^d) mice were lethally irradiated (8.5 Gy) on day -1 and transplanted with 3.5×10^6 TCD-BM with or without 1×10^6 splenic naïve $CD8^+$ T cells from WT (*Pgd*$^{fl/fl}$) or 6PGD deficient (*Pgd*$^{fl/fl}$*Cd4*^{Cre}) C57BL/6 (H-2^b) mice on day 0. Body weight loss (A) and clinical GvHD scores (B) are presented as mean ± SEM. N=5 mice per group. Data representative of one independent experiment. The Two-way ANOVA was used to analyze statistical significance among groups. Survival data (C) are presented as percent survival (Log-rank Mantel-Cox test).

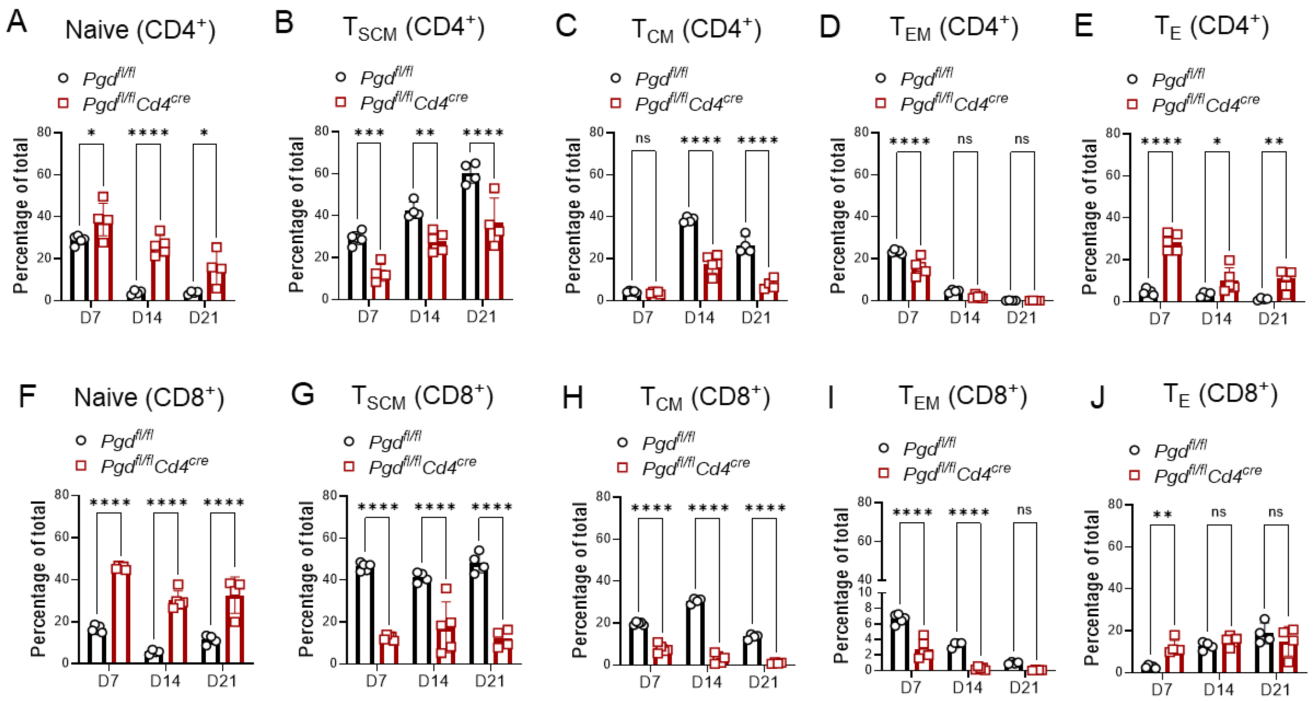

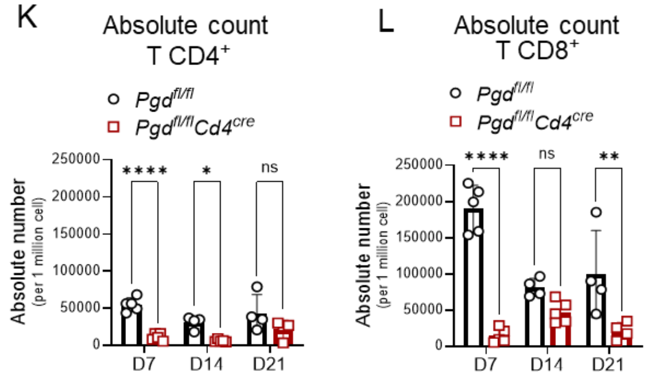

M

Subset	Markers
Naïve	CD45RA+ CCR7+ CXCR3- CD95- CD122-
T_{SCM}	CD45RA+ CCR7+ CXCR3+ CD122+ Sca1+
T_{CM}	CD45RA- CCR7+ CD95+ CD122+
T_{EM}	CD45RA- CCR7- CD95+ CD122+
T_E	CD45RA+ CCR7- CXCR3- CD95+ CD122+

Figure S7: T cell activation status in spleen, shown as percentage of total.

(A-J) Allo-HCT was performed as in Figure 2, and T cell subsets were evaluated by flowcytometry on day +7, +14, and +21 as in Figure 2A-J. The T cell subsets are shown as percentages of total $CD4^+$ (A-E) and total $CD8^+$ (F-J) cells. N = 4-5 mice per group. The Two-way ANOVA was used to analyze statistical significance among groups.

(K-L) After Allo-HCT as per A-J, T cell frequencies were examined on day +7, +14, and +21 as in Figure 2K-L. The frequencies of total $CD4^+$ (K) and per total $CD8^+$ (L) cells per million of splenocytes are given. N = 4-5 mice per group. The Two-way ANOVA was used to analyze statistical significance among groups.

(M) The table demonstrates the list of markers used to identify T cell subsets.

Data are shown as Mean ± SEM. *$p < 0.05$; **$p < 0.01$; ***$p < 0.001$; ****$p < 0.0001$.

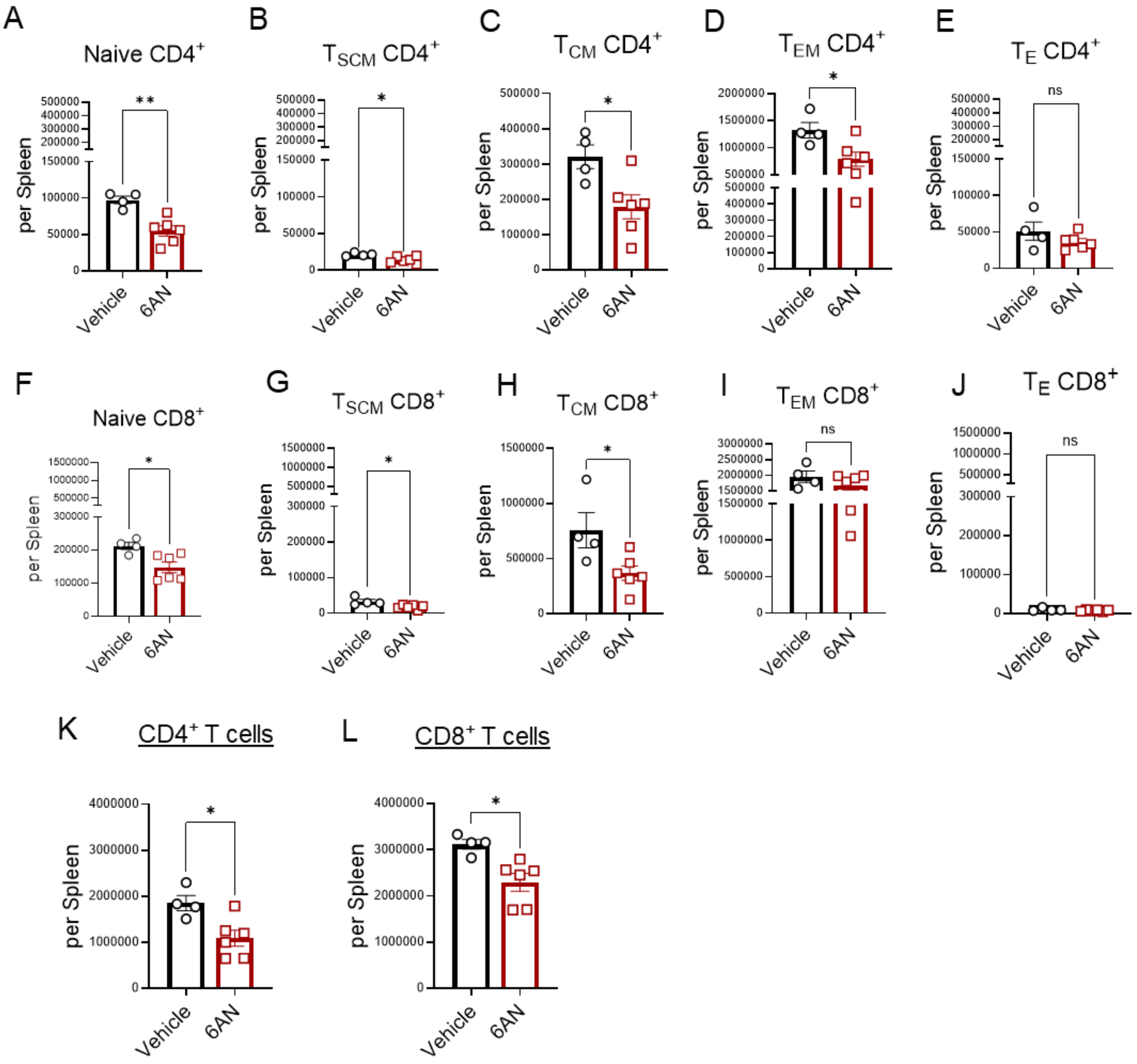

Figure S8: 6AN, a small molecule inhibitor of 6PGD suppresses T cell expansion in a xenogeneic GvHD model.

(A-L) NSG mice were sublethally irradiated (2.5 Gy) on day -1 and transplanted i.v. with 2×10^6 human PBMCs on day 0. Mice were injected with 1 mg/kg 6AN or vehicle (1% DMSO) every two days (intraperitoneally: i.p.). Spleen T cell phenotyping was evaluated on day +14 post transplantation. The frequencies of $CD4^+$ T cells subsets as naïve (A), stem cell memory (B), central memory (C), effector memory (D), and effector (E) were detected per spleen. The frequencies of $CD8^+$ T cells subsets as naïve (F), stem cell memory (G), central memory (H), effector memory (I), and effector (J) were determined per spleen. The absolute numbers of $CD4^+$ (K) and $CD8^+$ (L) T cells were calculated per spleen. N = 4-6 mice per group. The Student's t test was used to analyze statistical significance between 2 groups.

Data are shown as Mean ± SEM. *$p < 0.05$; **$p < 0.01$; ***$p < 0.001$; ****$p < 0.0001$.

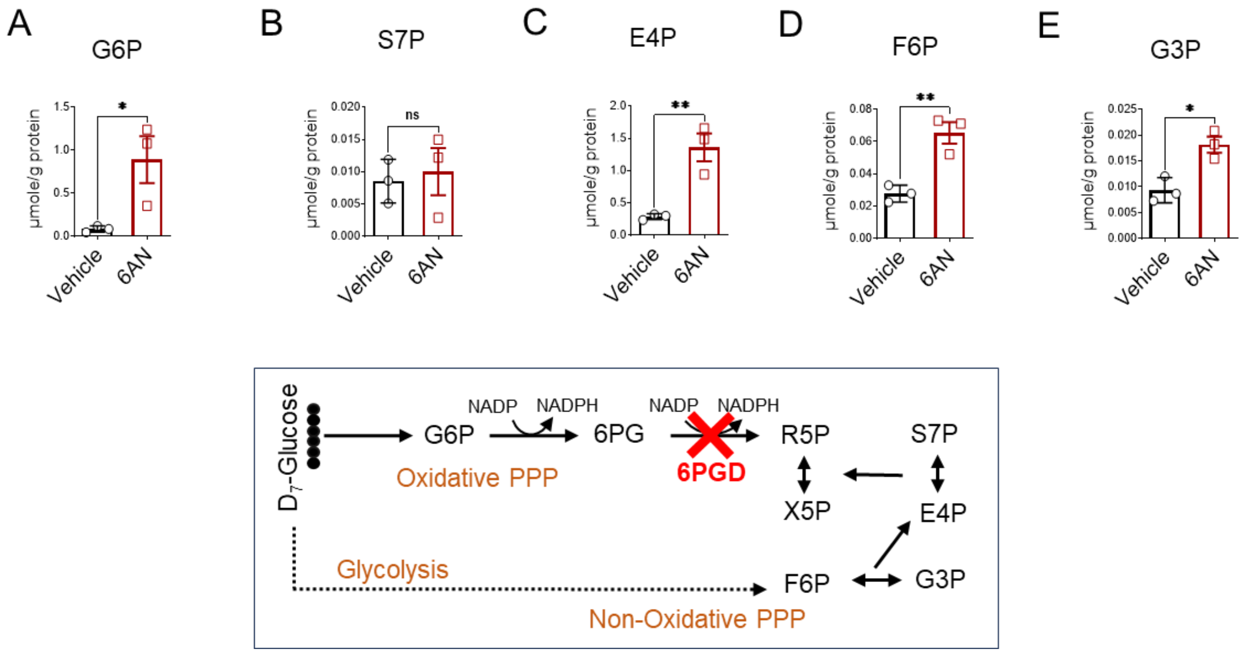

Figure S9: 6PGD blockade in T cells activates non-oxidative PPP as a compensatory mechanism.

(A) Splenic naïve T cells were isolated and examined for tracing study according to Figure 4A. The isotope-labeling of metabolites from glucose into the oxidative PPP and non-oxidative PPP at the 8 hr. timepoint is shown. Significant accumulation of 6PG indicate effective blockade of 6PGD enzyme. The buildup of R5P, the substrate of 6PGD enzyme demonstrate activation of non-oxidative PPP to generate the R5P required for nucleotide biosynthesis. Abbreviations: G6P: glucose-6-phosphate; 6PG: 6-phosphogluconate; R5P: ribose-5-phosphate; S7P: sedoheptulose-7-phosphate; E4P: erythrose-4-phosphate; G3P: glyceraldehyde-3-phosphate; F6P: fructose-6-phosphate. N = 3 datapoints per group. Data representative of two independent experiments. The Student's t test was used to analyze statistical significance between 2 groups.

Data are shown as Mean ± SEM. *$p < 0.05$; **$p < 0.01$.

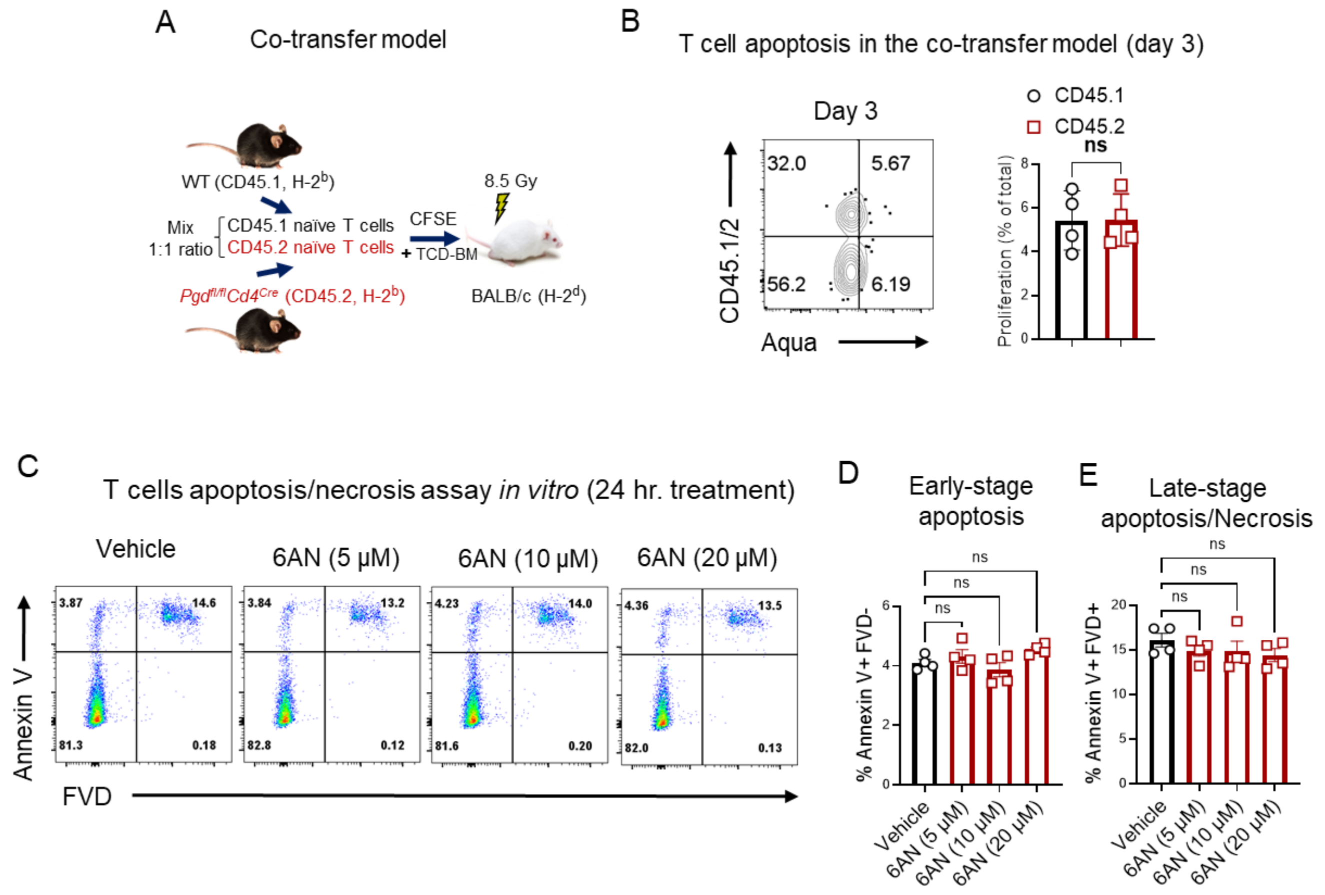

Figure S10: 6PGD blockade does not induce significant T cell apoptosis both *in vitro* and *in vivo*.

(A-B) Co-adaptive transfer model was generated as in Figure 2P. Percentage of dead cells was determined on day +3 by Aqua staining in splenic CD45.1^{+} and CD45.2^{+} T cells. N = 4-5 mice per group. One-way ANOVA was used to determine statistical differences between groups.

(C-E) Splenic and lymph node T cells were isolated from WT C57BL/6 mice and activated *in vitro* with plate bound anti-CD3 and anti-CD28 (10 μg/ml each) plus rmIL-2 (100 ng/ml) in presence of vehicle of 6AN (5-20 μM) for 72 hrs. The early and late apoptosis rates were determined by Annexin V staining. N=6 datapoint per group. Data representative of two independent repeats. The one-way ANOVA was used to analyze statistical significance between groups.

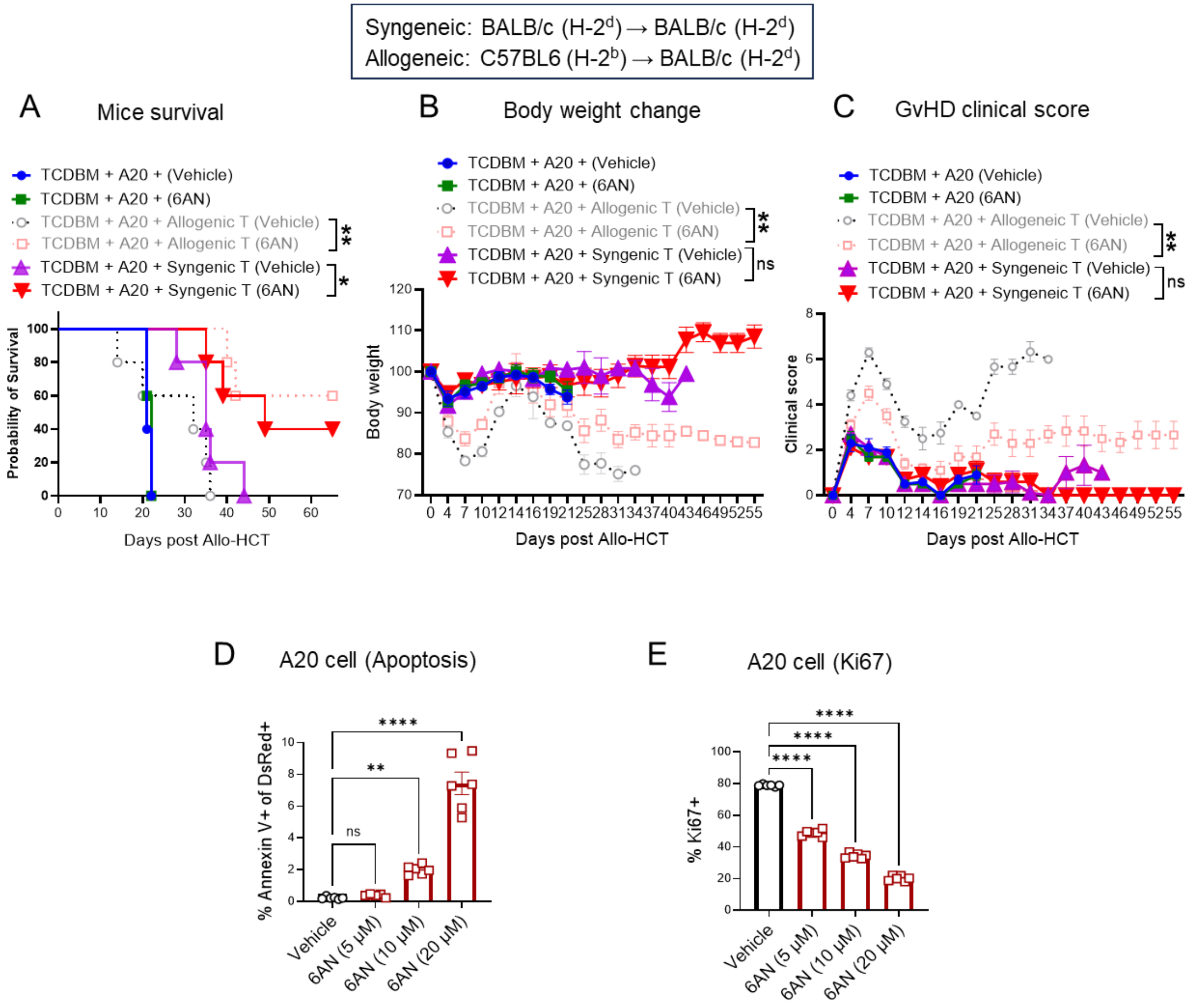

Figure S11: 6AN inhibitor prevents tumor growth and increases mice survival in syngeneic GvT model.

(A-C) BALB/c (H-2^d) mice were lethally irradiated (8.5 Gy) on day -1 and transplanted i.v. with 3.5x10^6 TCD-BM with or without 0.2x10^6 splenic naïve T cells from wild type BALB/c (H-2^d) mice plus 0.1x10^6 A20-luc$^+$ tumor cells on day 0. Mice were injected with 0.5 mg/kg 6AN or vehicle (1% DMSO) daily (intraperitoneally: i.p.). 6AN treatment resulted in lower tumor growth determined by increased mice survival (A), without change in mice body weight (B) and detectable clinical GvHD score (C). Data was measured and presented as mean ± SEM. Data representative of one independent experiment. Survival was examined by Log-rank Mantel-Cox test, and body weight change and clinical GvHD score by the Two-way ANOVA. N = 5 mice per group.

(D-E) Direct effect of 6AN on A20 tumor cells were measured *in vitro*. The cultured A20 cell line was exposed to increasing concentrations of 6AN (5-20 μM) or vehicle (DMSO) for 48 hrs. Increase in apoptosis rare was detected by Annexin V staining (D) and reduced proliferation was measured by Ki67 staining (E) in flowcytometry. Data was measured and presented as mean ± SEM. N = 6 mice per group. Data representative of two independent experiments. One-way ANOVA was used to determine statistical differences between groups.

Data are shown as Mean ± SEM. *$p < 0.05$; **$p < 0.01$; ***$p < 0.001$; ****$p < 0.0001$.
