# Supplementary material for: Targeting the pentose phosphate pathway mitigates graft-versus-host disease by rewiring alloreactive T cell metabolism
Source: JCI Insight. 2025 Dec 8;10(23):e192774. doi: 10.1172/jci.insight.192774 (PMC12890487; doi:10.1172/jci.insight.192774)
Supplement: Unedited blot and gel images [file jciinsight-10-192774-s152.pdf]

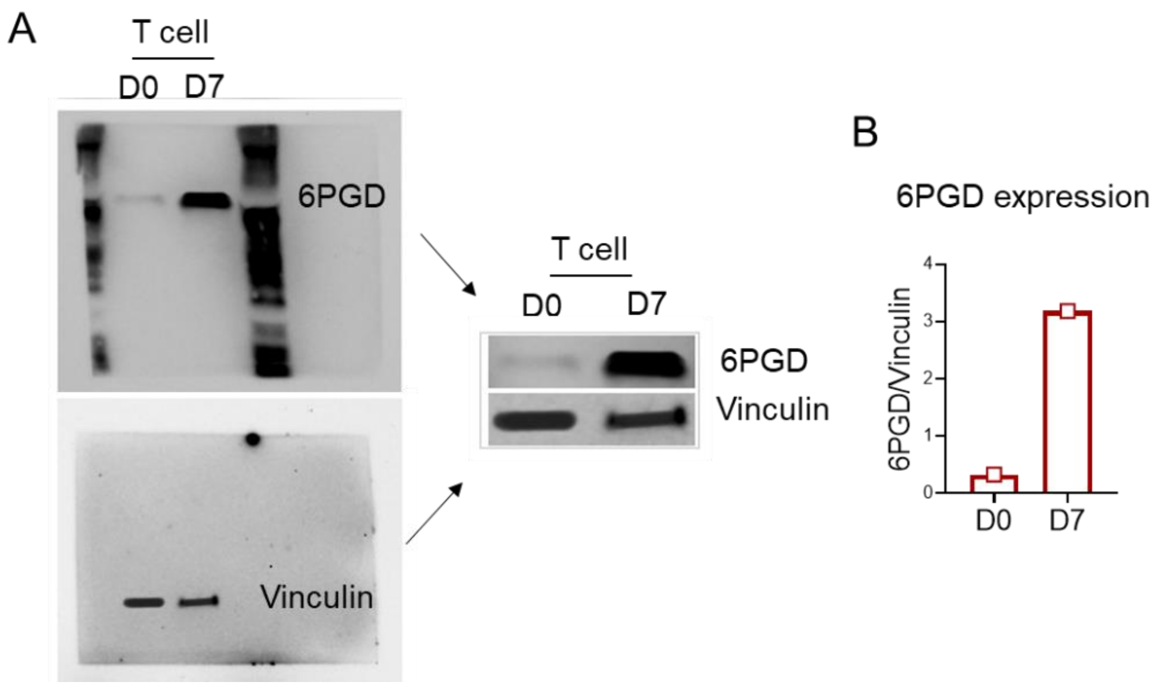

6PGD expression is enhanced in T cells during GvHD development.

(A-B) BALB/c (H-2^d) mice were lethally irradiated (8.5 Gy) on day -1 and transplanted intravenously with 3.5x10^6 TCD-BM with or without 0.2x10^6 splenic naïve T cells from WT C57BL/6 (H-2^b) mice on day 0. Expression of 6PGD was evaluated in donor T cells at the time of transplant (D0) compared to T cells isolated from recipient spleens on day +7 (D7) post transplantation by western blot analysis. Vinculin expression served as the control. Expression levels of 6PGD was calculated based on vinculin band expression.
